# Conclusive Insight into the Coordination Complexes of a Flexible Bis(β‐diketonato) Ligand and Their Phase‐Dependent Structure: A Multi‐Technique Approach

**DOI:** 10.1002/chem.202500697

**Published:** 2025-05-13

**Authors:** Manuel Imperato, Alessio Nicolini, Olga Mironova, Enrico Benassi, Nicola Demitri, Lara Gigli, Adele Mucci, Andrea Cornia

**Affiliations:** ^1^ Dipartimento di Scienze Chimiche e Geologiche e UdR INSTM Università degli Studi di Modena e Reggio Emilia via G. Campi 103 Modena 41125 Italy; ^2^ Dipartimento di Scienze Fisiche Informatiche e Matematiche Università degli Studi di Modena e Reggio Emilia via G. Campi 213/A Modena 41125 Italy; ^3^ Department of Natural Sciences Novosibirsk State University Pirogova St. 1 Novosibirsk 630090 Russia; ^4^ Elettra‐Sincrotrone Trieste S.C.p.A. Basovizza Trieste 34149 Italy; ^5^ Present address: Dipartimento di Scienze Fisiche Informatiche e Matematiche Università degli Studi di Modena e Reggio Emilia via G. Campi 213/B Modena 41125 Italy

**Keywords:** chelates, dosy, isomerization, nmr spectroscopy, x‐ray diffraction

## Abstract

Multichelating ligands with nuclear spin‐free donor atoms are of particular interest for creating stable electronic spin qubits based on paramagnetic transition metal ions. We recently focused on the coordinating ability of the bis(β‐diketonato) ligand bdhb^2−^, featuring two “acac” moieties connected through a 1,3‐phenylene bridge (H_2_bdhb = 1,3‐bis(3,5‐dioxo‐1‐hexyl)benzene). The two crystalline complexes of bdhb^2−^ so far isolated and structurally characterized, namely [(VO)_2_(bdhb)_2_] (**1**) and [Co_2_(bdhb)_2_(py)_4_] (**2**), are dimeric and contain bridging bdhb^2−^ ligands; however, they become mononuclear and quasi‐macrocyclic in organic solution. To investigate this unique structural isomerism by high‐resolution ^1^H NMR spectroscopy, we have now synthesized a diamagnetic Zn^2+^ analogue of **1** and **2**, namely [Zn_2_(bdhb)_2_(py)_2_] (**3**). Although both **2** and **3** are dimeric and contain the same ligands, **3** features only one pyridine molecule per metal ion, whose coordination geometry is square pyramidal rather than tetragonally elongated octahedral. The ESI‐MS spectra of **3** in THF and CH_2_Cl_2_ contain peaks from both monomeric and dimeric species. However, molecular weight determinations by DOSY and conformational studies based on *J*‐coupling analysis and DFT calculations conclusively prove the rearrangement of **3** into quasi‐macrocyclic monomers in THF‐*d*_8_ and CD_2_Cl_2_ solution at room temperature.

## Graphical abstract

Complexes of flexible bis(β‐diketonato) ligand bdhb^2−^ are dimeric in the crystalline state but rearrange to monomeric in organic solution. The transformation, first detected on paramagnetic VO^2+^ and Co^2+^ derivatives, is conclusively proved through the preparation of diamagnetic [Zn_2_(bdhb)_2_(py)_2_] and its investigation by high‐resolution ^1^H NMR spectroscopy in THF‐*d*_8_ and CD_2_Cl_2_.

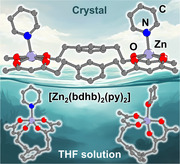

## 1 Introduction

In recent years, the role of the molecular approach^[^ ^1^, ^2^, ^3^, ^4^, ^5^, ^6^, ^7^, ^8^, ^9^ ^]^ to creating units of quantum information has significantly expanded as a viable alternative to the more widespread top‐down methods based on solid‐state defects in inorganic materials.^[^ ^10^, ^11^ ^]^ This has caused a burst of attention to paramagnetic complexes of first‐row transition metals as adjustable electronic spin qubits with potentially long coherence times. In general, structural robustness has a positive effect on coherence lifetime by reducing conformational flexibility and minimizing interactions with the surrounding environment, two factors that often contribute to decoherence.^[^ ^12^, ^13^, ^14^ ^]^ Beneficial are also a nuclear spin‐free environment around the metal and a weak spin‐orbit coupling. Coexistence of all these features explains the overly long phase memory times (*T*_m_) displayed by vanadium(IV) dithiolene complexes [VO(β‐C_3_S_5_)_2_]^2−[^ ^15^ ^]^ and [V(C_8_S_8_)_3_]^2−^,^[^ ^16^ ^]^ with the record value *T*_m_ = 0.7 ms measured on a frozen solution of the latter in the nuclear spin‐free solvent CS_2_ at 10 K.^[^ ^16^ ^]^

Macrocyclic ligands like porphyrins^[^ ^17^, ^18^, ^19^ ^]^ and phthalocyanines^[^ ^13^, ^20^ ^]^ are widely used platforms to access enhanced structural stability and rigidity, although the nuclear spin of N donor atoms can adversely affect coherence properties. Consequently, there is still wide room for improvement in the design of stable molecular spin qubits containing nuclear spin‐free macrocyclic ligands.

**Figure 1:**
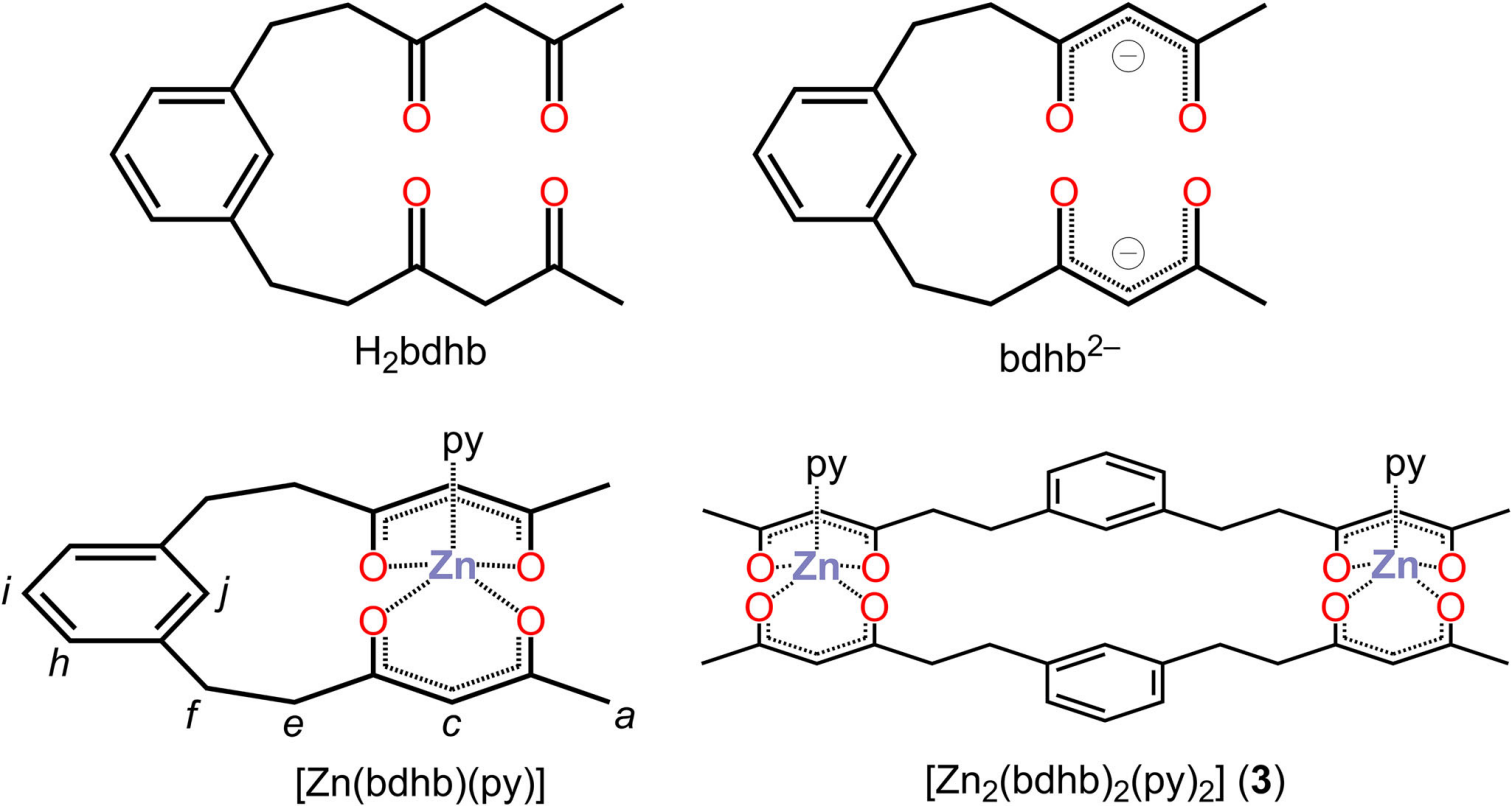
Structures of pro‐ligand H_2_bdhb, ligand bdhb^2−^, monomeric [Zn(bdhb)(py)] (with the atom labeling scheme used in the discussion of NMR spectra), and dimeric [Zn_2_(bdhb)_2_(py)_2_] (**3**) complexes.

In this context, we recently attempted the synthesis of macrocyclic pro‐ligand 3,5,16,18‐tetraoxo[7.7]metacyclophane,^[^ ^21^, ^22^ ^]^ as it forms neutral complexes with divalent cations and contains nuclear spin‐free donors. So far, we did not succeed in obtaining the target macrocycle in synthetically useful yields, and we shifted our attention to the quasi‐macrocyclic intermediate 1,3‐bis(3,5‐dioxo‐1‐hexyl)benzene (H_2_bdhb) displayed in Figure 1. [(VO)_2_(bdhb)_2_] (**1**)^[^ ^23^ ^]^ and [Co_2_(bdhb)_2_(py)_4_] (**2**)^[^ ^24^ ^]^ were the first metal complexes of bdhb^2−^ authenticated by X‐ray diffraction. In the crystalline state, **1** and **2** are dimeric and contain bridging bdhb^2−^ ligands, but ^1^H DOSY NMR and ESI‐MS spectrometry indicate that they isomerize to quasi‐macrocyclic monomers in solution. Interestingly, a frozen solution of **1** in toluene‐*d*_8_/CD_2_Cl_2_ displayed a phase memory time reaching 13 µs at 10 K, which makes this vanadyl complex a good spin‐coherent building block in quantum technologies.^[^ ^23^ ^]^ For comparison, in the same conditions, [VO(dpm)_2_] (Hdpm = dipivaloylmethane) exhibits a phase memory time below 3 µs.^[^ ^25^ ^]^

Since NMR structural investigations on paramagnetic species are complicated by significant signal broadening and shifting, we have searched for a definitive confirmation of the described structural rearrangement by designing a diamagnetic dizinc(II) analogue of **1** and **2**. The isolated complex, [Zn_2_(bdhb)_2_(py)_2_] (**3**), is not isostructural with **2** because of the presence of a single py ligand per metal ion. However, the diamagnetism of **3** has allowed us to investigate its structure in solution by fully exploiting the potential of ^1^H NMR techniques to provide information on molecular weight (DOSY) and ligand's conformation (*J*‐coupling analysis). The results, complemented by DFT calculations, provide conclusive evidence of the proposed dual structure in the crystalline state and in solution.

## 2 Results and Discussion

### 2.1 Synthesis

The H_2_bdhb pro‐ligand (Figure 1) was prepared by modification of the procedure first reported by Alberts and Cram,^[^ ^21^, ^22^ ^]^ as was described before.^[^ ^24^ ^]^ A white solid with composition Zn(bdhb)(H_2_O)_2_ (**3′**) was isolated by reacting H_2_bdhb with Zn(OAc)_2_·2H_2_O in dry MeOH under an inert atmosphere. Compound **3′** is highly soluble in THF, but the addition of an excess of pyridine to the solution causes immediate precipitation. A more controlled procedure involves layering a THF solution of **3′** with an *n*‐hexane/pyridine mixture (6:1 molar ratio of pyridine to Zn), which reproducibly affords colorless, needle‐like microcrystals of the dimeric complex [Zn_2_(bdhb)_2_(py)_2_] (**3**) (see Figure S1). Unfortunately, these crystals were too small and fragile for a single‐crystal X‐ray diffraction (SCXRD) investigation using conventional equipment, but suitable for synchrotron SCXRD, which identified them as triclinic crystal phase **3*a*** (Note: we label the crystal phases with the crystal family symbol in Pearson notation^[^ ^26^ ^]^). By slow concentration of the mother liquor from a crystallization test, a different orthorhombic polymorph **3*o*** occasionally crystallized in a form suitable for laboratory SCXRD (Figure S2). Individuals of the same crystal phase were also picked out from bulk samples comprising mainly **3*a***. However, they were present in very low amounts and escaped detection by powder X‐ray diffraction (PXRD), as shown below. For this reason, the bulk samples used for solid‐state characterizations are labeled simply as **3*a***.

Despite the large excess of pyridine used in the synthesis, all characterization data concurrently indicate that **3** contains a single py ligand per metal ion. Similarly, using acac^−^ ligands and a Zn:pyridine molar ratio of 1:2^[^ ^27^ ^]^ only the five‐coordinate complex [Zn(acac)_2_(py)] (**4**) could be reproducibly isolated as a stable compound.^[^ ^28^, ^29^, ^30^, ^31^ ^]^ According to Graddon et al.,^[^ ^32^ ^]^ [Zn(acac)_2_(py)_2_] can be obtained by recrystallization of **4** from pure pyridine, but it is very unstable due to the rapid loss of pyridine and transforms back to the monopyridine adduct. These authors also explained the greater stability of [Co(acac)_2_(py)_2_] versus **4** based on the crystal‐field stabilization energy, which is large for a high‐spin cobalt(II) ion but is equal to zero for the closed‐shell zinc(II) ion.

The solid‐state FT‐IR spectra of **3′** and **3*a*** are more resolved than that of the pro‐ligand H_2_bdhb (Figures S9 and S10). The intense C═O and C═C stretching bands of H_2_bdhb between 1724 and 1587 cm^−1^ undergo a red shift of approximately 100 cm^−1^ upon coordination. Differences lie in the presence of the broad OH stretching band of water molecules centered at about 3300 cm^−1^ in **3′**, and in the additional signals of pyridine in **3*a***. In particular, the bands at 638 and 419 cm^−1^ (640 and 425 cm^−1^ in **4** ^[^ ^28^, ^33^ ^]^) are assigned to the in‐plane and out‐of‐plane bending of the pyridine ring, respectively. The Zn–O stretching bands (558 and 425 cm^−1^ in **4**)^[^ ^28^, ^33^ ^]^ appear at 563 and 418 cm^−1^ in **3′**, and at 559 and 419 cm^−1^ in **3*a*** (the latter overlapping with the out‐of‐plane bending band of pyridine).

### 2.2 X‐ray Crystallography

**Figure 2:**
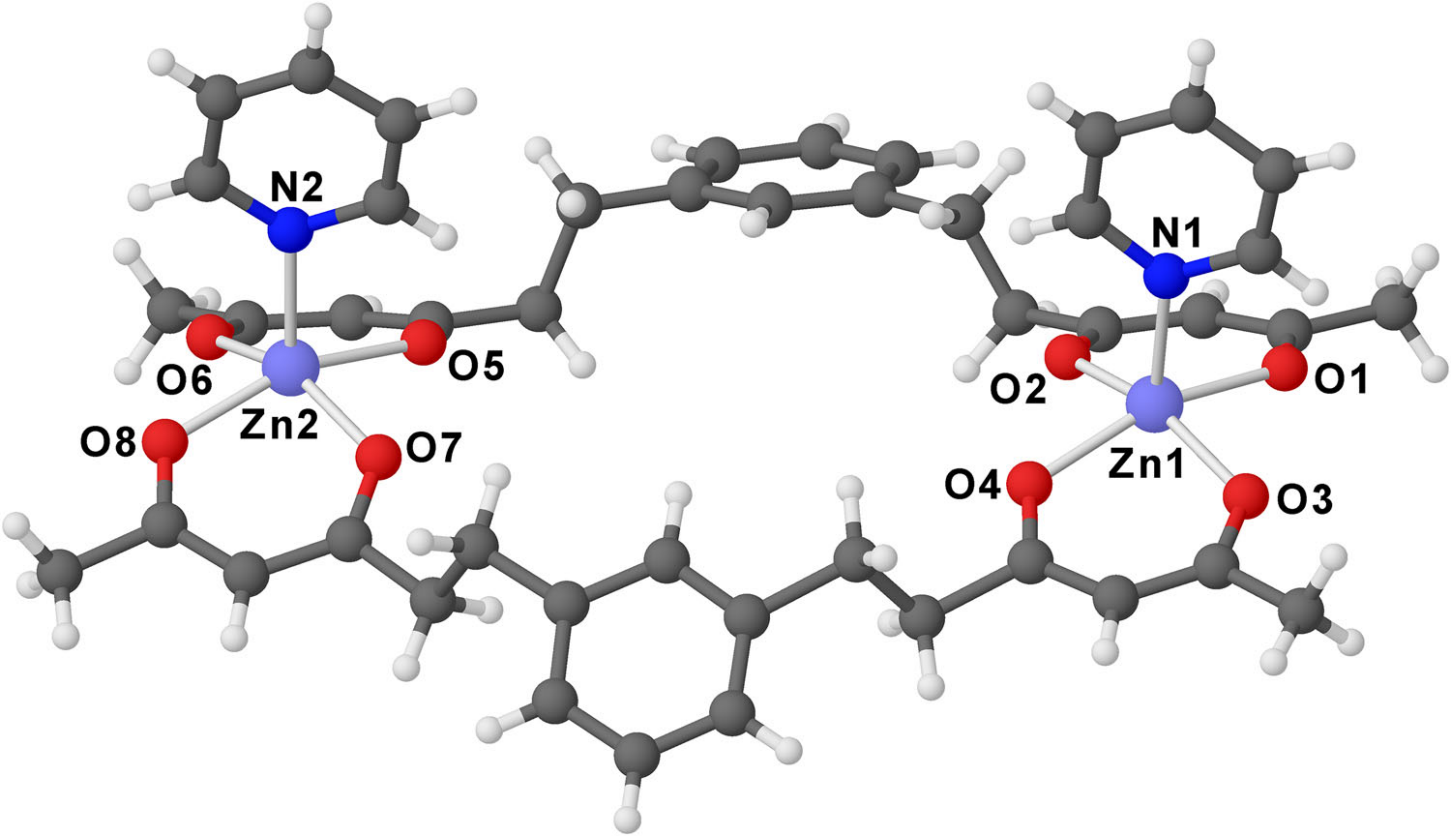
Structure of dimeric molecules in **3*o*** with the atom labeling scheme (color code: C = dark grey, H = white, O = red, N = blue, Zn = light blue).

Crystal data and refinement parameters for **3*o*** and **3*a*** are presented in Table S1, and interatomic distances and interbond angles are summarized in Table 1. We base our structural description on **3*o***, which gave better experimental data and lower *R*‐indices. An SCXRD analysis performed at room temperature revealed that **3*o*** crystallizes in the orthorhombic *Fddd* space group with a large unit cell (*Z* = 32, *V* ∼ 35,500 Å^3^). As found in **1** ^[^ ^23^ ^]^ and **2**,^[^ ^24^ ^]^ the crystal lattice of **3*o*** contains dimeric molecules, in which two bdhb^2−^ ligands embrace two zinc(II) ions at 11.4702(17) Å from each other (Figures 2 and S3). However, while the dimers in **1** and **2** are centrosymmetric, the dimeric molecules in **3*o*** are non‐centrosymmetric. Each metal center is pentacoordinated, featuring a square pyramidal geometry with one py ligand in apical position (Zn─N = 2.090–2.113 Å).

**Table 1:** Selected interatomic distances (Å) and interbond angles (°) in **3*o*** and **3*a***, with estimated standard deviations in parentheses.

	**3*o***	**3*a***		**3*o***	**3*a***
Zn1⋯Zn2	11.4702(17)	11.392(5)	Zn2─N2	2.113(7)	2.087(10)
Zn1─O1	2.031(6)	2.036(10)	O1─C2	1.239(9)	1.269(16)
Zn1─O2	2.011(6)	1.985(10)	O2─C4	1.240(9)	1.294(17)
Zn1─O3	2.037(6)	2.008(10)	O3─C20	1.295(10)	1.259(17)
Zn1─O4	2.045(6)	2.049(10)	O4─C22	1.301(11)	1.286(16)
Zn1─N1	2.090(7)	2.066(12)	O5─C15	1.281(10)	1.274(17)
Zn2─O5	2.031(6)	2.021(9)	O6─C17	1.285(10)	1.283(16)
Zn2─O6	2.041(6)	2.005(10)	O7─C33	1.255(9)	1.251(16)
Zn2─O7	2.043(6)	1.991(11)	O8─C35	1.236(10)	1.240(17)
Zn2─O8	2.009(6)	2.022(10)	–	–	–
N1─Zn1─O1	93.8(3)	98.1(4)	N2─Zn2─O5	98.0(3)	95.7(4)
N1─Zn1─O2	105.5(3)	107.4(4)	N2─Zn2─O6	106.5(3)	106.6(4)
N1─Zn1─O3	94.7(3)	102.7(4)	N2─Zn2─O7	99.0(3)	100.5(4)
N1─Zn1─O4	105.0(3)	97.6(4)	N2─Zn2─O8	103.0(3)	97.7(4)
O1─Zn1─O2	90.2(2)	89.4(4)	O5─Zn2─O6	89.0(2)	89.8(4)
O1─Zn1─O3	86.6(2)	87.0(4)	O5─Zn2─O7	85.8(3)	88.6(4)
O2─Zn1─O4	88.0(2)	86.5(4)	O5─Zn2─O8	158.9(3)	166.5(4)
O3─Zn1─O4	88.5(2)	88.9(4)	O6─Zn2─O7	154.4(3)	152.9(4)
O1─Zn1─O4	160.9(3)	164.2(4)	O6─Zn2─O8	87.2(2)	85.6(4)
O2─Zn1─O3	159.7(3)	149.9(4)	O7─Zn2─O8	88.7(2)	89.8(4)

The O donors making up the base of the pyramid (Zn─O = 2.01–2.04 Å) are almost coplanar, with root‐mean‐square (RMS) deviations of 0.01 Å for O1, O2, O3, O4, and 0.04 Å for O5, O6, O7, O8. The zinc atoms are shifted out of this plane by 0.35–0.41 Å toward the single apical py ligand. By contrast, in dicobalt(II) complex **2**, the coordination geometry is tetragonally elongated octahedral, with the two py ligands in the axial positions, and each metal center is virtually coplanar with its four O donors.^[^ ^24^ ^]^ The py ligands at Zn1 and Zn2 adopt a *cis* configuration and have almost parallel mean planes (6.3°). They are tilted in the same direction, one toward the molecule's center, and the second toward the outside (Figure S4). As a result, the angles between the Zn1─N1 and Zn2─N2 vectors and the normals to the basal planes take on values of 9.9 and 6.2°, respectively.

In complexes **1**–**3**, the bdhb^2−^ ligands are unfolded in bridges connecting two metal centers, but their conformation is profoundly different (Figure S5). Although the ethylene bridges always display an antiperiplanar conformation, their orientation with respect to the phenylene moieties is not the same. In divanadyl complex **1**, the ethylene bridges are on opposite sides of the phenylene ring, and the ligand's skeleton takes the form of a ladder, resulting in idealized *C*_2h_ point‐group symmetry (in Schönflies notation) with the V═O moieties lying on the mirror plane.^[^ ^23^ ^]^ In both the dicobalt(II) (**2**)^[^ ^24^ ^]^ and dizinc(II) (**3**) complexes, the ethylene bridges are on the same side of the phenylene ring. However, while the phenylene rings in centrosymmetric **2** are parallel and placed on opposite sides of the RMS plane built through the Co and O atoms, in **3** they are at an angle of 49.6° from each other and lie on the same side of the four β‐diketonato fragments, toward the py ligands. By consequence, complex **2** conforms to idealized *C*_2h_ symmetry, with the twofold axis going through the Co atoms, while complex **3** has idealized *C*_2v_ symmetry, with the twofold axis normal to the average molecular plane and the Zn and N atoms on a symmetry plane. Such different structural motifs are presumably caused by the different nature of the metals and the filling of their coordination sphere with coordinating solvents.

As shown in Figure S6, the unit cell of **3*o*** contains eight solvent‐accessible voids of 255 Å^3^, with 90 electrons per void. Since THF and *n*‐hexane, the two solvents used for crystallization, have 40 and 50 electrons per molecule, respectively, the data are compatible with the presence of ∼2 solvent molecules per void, that is, ∼16 solvent molecules per unit cell. Considering that *Z *= 32, the compound can be formulated as **3**·(THF)*_n_* (*n*‐hexane)*_m_* with *n*+*m *= 0.5. Only residual solvent traces were however detected by ^1^H NMR spectroscopy after dissolving the microcrystalline bulk product in CD_2_Cl_2_ (Figure S11). Concurrently, combustion analysis on the same bulk material never showed the presence of residual solvent. Furthermore, the experimental PXRD pattern and the pattern calculated from the single‐crystal structure of **3*o*** evidently do not match (Figure S7). The reason is that the bulk microcrystalline material contains only minor amounts of **3*o***, while the by far dominant phase is **3*a***. SCXRD data on this second phase were collected using synchrotron radiation. The analysis showed that **3*a*** is triclinic (space group *P* $\bar{1}$) and solvent‐free. Its molecular structure is, however, almost superimposable with that of **3*o*** (calculated RMS deviation of atomic positions = 0.362 Å), as shown in Figure S8. One difference is that the dihedral angle between the two pyridine rings is significantly larger in **3*a*** than in **3*o*** (23.3° versus 6.3°). Rewardingly, the simulated powder diffractogram of **3*a*** is in good agreement with the measured pattern (Figure S7).

### 2.3 Solution Structure

**Figure 3:**
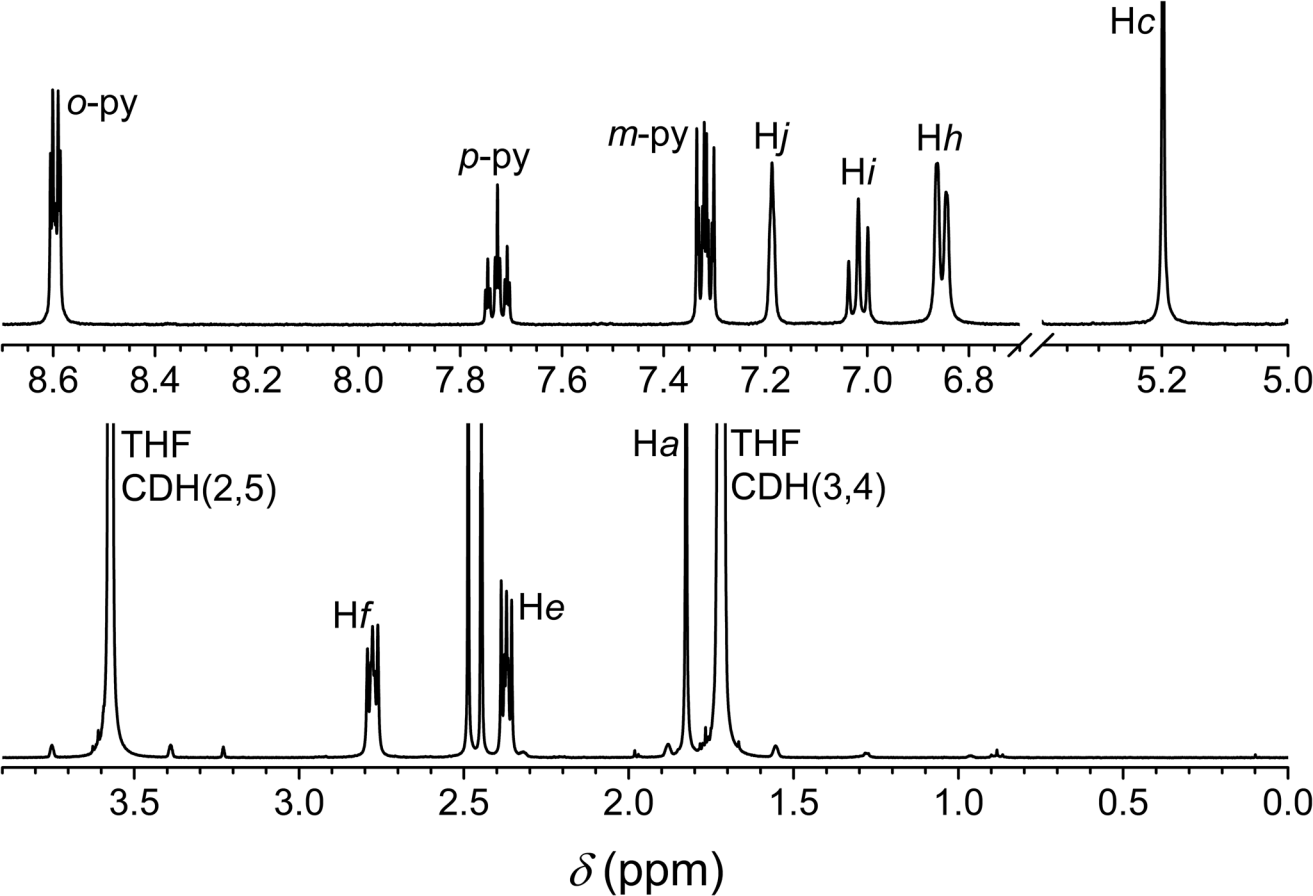
^1^H NMR spectrum of **3** in THF‐*d*_8_ (400.13 MHz, 298 K); *δ* = 2.49 (s; H_2_O), 2.45 ppm (t; HOD); processing parameters [TopSpin 4.3.0^[^ ^34^ ^]^]: SI = TD, LB = 0.30 Hz.

Owing to its two β‐diketonic branches, H_2_bdhb exists in three detectable tautomeric forms in organic solution, as established by NMR spectroscopy.^[^ ^24^ ^]^ Metal complexation requires deprotonation of each acac group and thus suppresses keto–enolic tautomerism, affording much simpler ^1^H NMR spectra. Furthermore, the diamagnetic properties of zinc(II) give access to high‐resolution spectra. The ^1^H NMR spectrum of **3** in THF‐*d*_8_ is displayed in Figure 3.

It shows ten distinct and well‐resolved signals with 2:1:2:1:1:2:2:4:4:6 relative integrated intensities (in order of decreasing *δ*), whose assignment is straightforward (see Figure 1 for the labeling scheme). Only seven of these signals, namely those at *δ* < 7.2 ppm, can be attributed to the coordinated bdhb^2−^ ligand, demonstrating that the latter maintains its twofold symmetry on the NMR timescale. The three remaining resonances at the lowest field (8.60, 7.73, and 7.32 ppm) arise from pyridine molecules. As a confirmation, compound **3′** dissolved in THF‐*d*_8_ (Figure S20) and acetone‐*d*_6_ only displays the seven well‐resolved ^1^H NMR signals of complexed bdhb^2−^, while the three resonances attributed to pyridine are missing.

The *δ* values of pyridine protons undergo a downfield shift of 0.06–0.08 ppm with respect to those of free pyridine in the same solvent (8.54, 7.65, and 7.25 ppm).^[^ ^35^ ^]^ This indicates that pyridine molecules are at least partially coordinated to the Zn^2+^ ions. The occurrence of fast chemical exchange between free and bound pyridine was confirmed by the diffusion coefficients (*D*) measured by ^1^H DOSY NMR spectroscopy.

**Figure 4:**
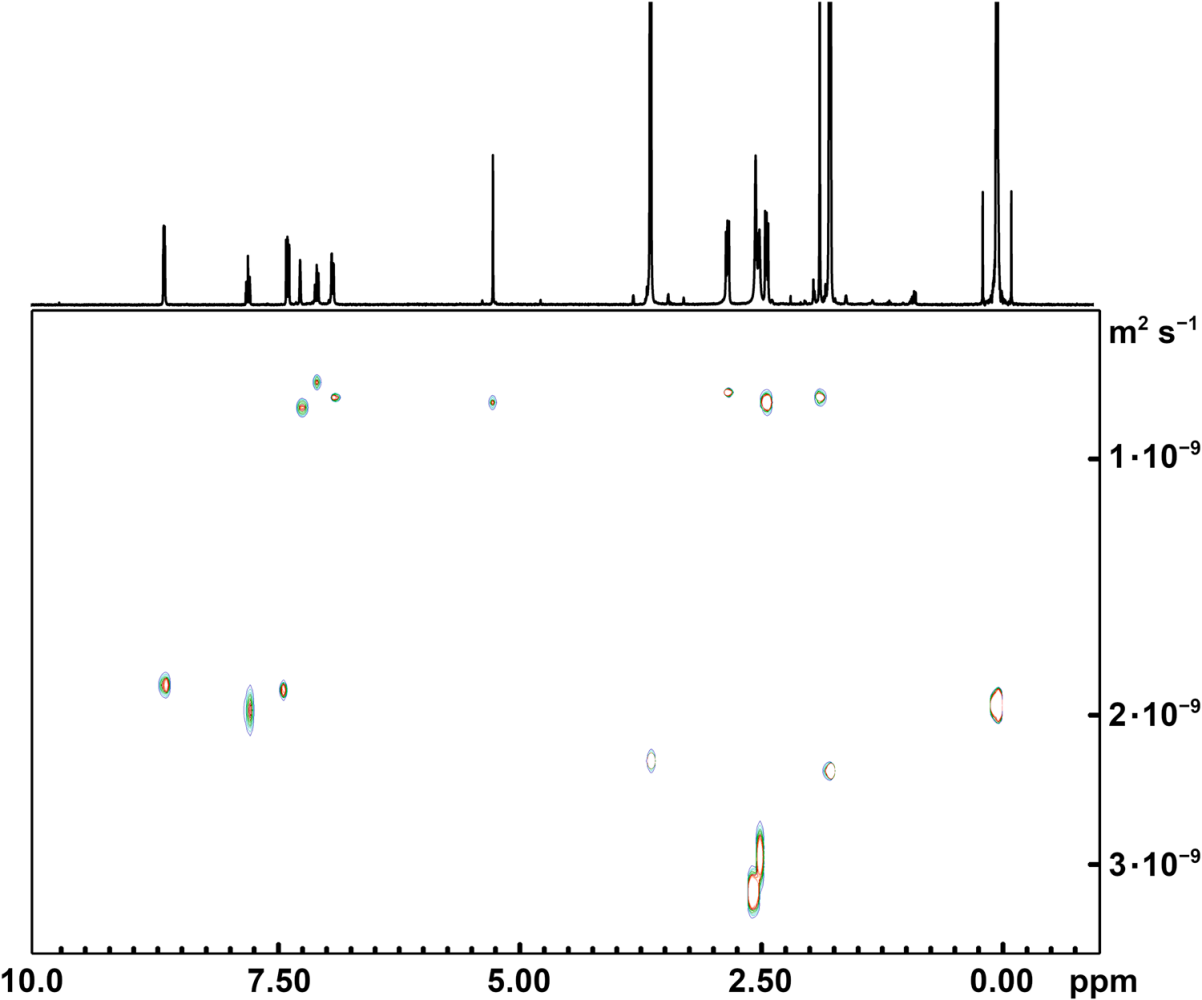
^1^H DOSY NMR spectrum of **3** in THF‐*d*_8_ (400.13 MHz, 298 K); *δ* = 2.49 (s; H_2_O), 2.45 (t; HOD), 0.00 ppm (s; TMS); processing parameters [TopSpin 4.3.0^[^ ^34^ ^]^]: SI = TD, LB = 0.30 Hz.

Figure 4 shows that the seven signals of coordinated bdhb^2−^ provide similar diffusion coefficients, which give an average *D*_bdhb_ = 8.7·10^−10^ m^2^ s^−1^ (all *D* values quoted herein are averaged over multiple protons). The diffusion coefficients associated with the three pyridine signals are also close to each other (*D*_py_ = 1.91·10^−9^ m^2^ s^−1^) and indicate that the py ligands diffuse faster than coordinated bdhb^2−^ but slower than free pyridine in THF‐*d*_8_ (2.29·10^−9^ m^2^ s^−1^). Assuming that *D*_py_ is a weighted average of the values for free and bound pyridine (the latter being equal to *D*_bdhb_), the percentages of free and bound pyridine are estimated as 73% and 27%, respectively.^[^ ^24^, ^36^, ^37^ ^]^ The molecular weight (*MW*) of the bdhb^2−^ complex was then estimated from *D*_bdhb_ using state‐of‐the‐art external calibration curves (ECCs), which yielded 417 ± 67 (ECC_DSE_) and 435 ± 113 (ECC_MERGE_) g mol^−1^ depending on the particular parameter set used.^[^ ^38^ ^]^ The calculated *MW*s for dimeric **3** and monomeric [Zn(bdhb)(py)] are 889.68 and 444.84 g mol^−1^, respectively. Therefore, the DOSY experiment establishes that **3** undergoes rearrangement to monomers in THF‐*d*_8_, as graphically represented in Figure S14. Note that pyridine and THF‐*d*_8_ have very similar *MW*s (79.10 and 80.16 g mol^−1^, respectively) and that replacing the py ligand by THF‐*d*_8_ would affect the overall *MW* only marginally. For a direct comparison, the DOSY analysis carried out on the H_2_bdhb pro‐ligand (*MW* = 302.36 g mol^−1^) in THF‐*d*_8_ gave *D*_bdhb_ = 9.7·10^−10^ m^2^ s^−1^ (Figure S13). This value is only 11% higher than that obtained for **3** and leads to estimated *MW*s of 345 ± 55 and 357 ± 91 g mol^−1^ using ECC_DSE_ and ECC_MERGE_ parameters, respectively.^[^ ^38^ ^]^ We conclude that the solvodynamic radii of H_2_bdhb and of the zinc(II) complex are similar, further demonstrating that **3** does not maintain its dimeric structure in THF‐*d*_8_ solution.

The DOSY analysis was also conducted in CD_2_Cl_2_, a non‐coordinating solvent, and similar conclusions were reached (Figures S11, S12, and S14).^[^ ^39^ ^]^ However, in this case, the percentage of bound pyridine was estimated to be as large as 76%. Concurrently, the signals of pyridine protons are downfield shifted by 0.06–0.17 ppm with respect to those of free pyridine in CD_2_Cl_2_ (8.59, 7.68, and 7.28 ppm).^[^ ^35^ ^]^ Hence, the deviation is significantly larger than in THF‐*d*_8_ (0.06–0.08 ppm). These results suggest that the large excess of THF‐*d*_8_, a coordinating solvent, may contribute to displacing pyridine from the zinc(II) coordination sphere. All DOSY data in THF‐*d*_8_ and CD_2_Cl_2_ are gathered in Table 2.

**Table 2:** Experimental *D* values (m^2^ s^−1^) for **3**, free H_2_bdhb and free pyridine in THF‐*d*_8_ and CD_2_Cl_2_, and estimated values of *MW* (g mol^−1^).

	THF‐*d*_8_			CD_2_Cl_2_		
	**3**	H_2_bdhb	py	**3**	H_2_bdhb^[^ ^23^, ^24^ ^]^	py^[^ ^24^ ^]^
*D*_bdhb_	8.7·10^−10^	9.7·10^−10^	–	9.4·10^−10^	1.2·10^−9^	–
*D*_py_	1.91·10^−9^	–	2.29·10^−9^	1.34·10^−9^	–	2.65·10^−9^
*MW* ^[^ ^a^ ^]^	417 ± 67	345 ± 55	–	521 ± 156	362 ± 105	–
*MW* ^[^ ^b^ ^]^	435 ± 113	357 ± 91	–	578 ± 187	391 ± 122	–

^[a]^ From ECC_DSE_ in Refs. [38, 39]

^[b]^ From ECC_MERGE_ in Refs. [38, 39]

The ESI‐MS spectra were recorded in positive‐ion mode on solutions of **3′** and **3** in CH_2_Cl_2_, THF, and their mixtures with MeCN (∼5:1 v/v) (Figures S16 and S17). The addition of MeCN provided a better ionization efficiency without significantly affecting the overall spectral pattern. These spectra demonstrate the presence of both [Zn(bdhb)] and [Zn_2_(bdhb)_2_] complexes, which are detected exclusively as adducts with alkali metal ions (Li^+^, Na^+^, K^+^). Intense peaks with a distribution pattern characteristic for Zn isotopes are observed in the areas corresponding to [Zn(bdhb)AM]^+^ and [Zn_2_(bdhb)_2_AM]^+^ (AM = Li, Na, K), with the species [Zn_2_(bdhb)_2_Na]^+^ giving the strongest signal in the spectrum (*m*/*z* = 753.1). Its fragmentation under ESI conditions results in a dominant peak with a poorly resolved isotopic pattern at *m*/*z* = 389.9, hence close to the position of [Zn(bdhb)Na]^+^. Notice that the positive‐ion ESI‐MS spectra of **2** in THF are largely dominated by the signals of cobalt(III) complexes [Co(bdhb)(py)]^+^ and [Co(bdhb)(py)_2_]^+^ rather than by the corresponding cobalt(II) adducts with alkali metal ions.^[^ ^24^ ^]^ We attribute this difference to the higher third ionization energy of Zn versus Co, which makes [Zn*_n_* (bdhb)*_n_*] complexes only detectable in the positive‐ion spectra after ligand protonation or formation of alkali metal cationic clusters.

In addition to this, a number of minor features attributed to purely organic species and to the ligand's alkali metal salts were detected in the positive‐ion ESI‐MS spectra. Notably, no species containing coordinated pyridine, THF, MeCN, or H_2_O were found in the spectra of **3′** and **3**. Upon addition of a large excess of pyridine to the CH_2_Cl_2_ solution of **3** (∼1:5 v/v), the peaks of Na and Li‐containing adducts fully persisted, while weak additional signals appeared (Figure S17); however, these signals could not be firmly attributed to pyridine‐containing species.

These observations confirm that the bdhb^2−^ ligand can form both monomeric and dimeric complexes with zinc(II) ions. Given the unequivocal results of DOSY analysis in THF‐*d*_8_ at room temperature, we conjecture that the higher ESI‐MS response of dimeric versus monomeric species may reflect either a structural change under ionization conditions, which imply the progressive removal of the solvent, or a higher stability of dimeric versus monomeric adducts with alkali metal ions.

The rearrangement of **1** ^[^ ^23^ ^]^ and **3** to quasi‐macrocyclic monomers in solution implies, as a necessary consequence, that no heterobimetallic complexes can be formed as a result of metal scrambling. These mixed‐metal dimeric species would contain one diamagnetic (Zn^2+^) and one paramagnetic (V^4+^) metal ion, and their ^1^H NMR spectra would be very different from those of both **1** and **3**. To check this point, we mixed approximately equimolar amounts of **1** and **3** in CD_2_Cl_2_ and analyzed the solution by ^1^H NMR spectroscopy over a time span of 7 months. The results, presented in Figure S15, show that the narrow peaks of the bdhb^2−^ ligands coordinated to zinc(II) are not affected by the presence of **1**. Instead, the pyridine proton signals of **3** undergo a very significant broadening with minimal alteration of their *δ* values, suggesting that pyridine molecules interact at least in part with the paramagnetic V^4+^ ions. This presumably occurs through the free coordination site *trans* to the oxo group^[^ ^40^ ^]^ and also explains the slightly modified chemical shifts exhibited by the broad resonances of the bdhb^2−^ ligands bound to the vanadyl groups. In conclusion, apart from the above‐described alterations caused by the chemical exchange of py ligands, **1** and **3** maintain their individual spectroscopic fingerprints in the mixed solution, and no heterobimetallic species are formed.

Finally, the highly resolved ^1^H NMR spectra of **3** in both THF‐*d*_8_ and CD_2_Cl_2_ allowed a detailed analysis of the second‐order multiplets arising from H*f* and H*e* (see labeling in Figure 1). The two signals could be accurately reproduced treating the ─CH_2_─CH_2_─ moiety as a simple AA'BB' spin system, where AA' and BB' represent the methylene protons H*f* and H*e*, respectively, with ^3^ *J*_AB_ = ^3^ *J*_A'B'_ and ^3^ *J*_AB'_ = ^3^ *J*_A'B_ (see Supporting Information for details). Symmetry equivalence within the A/A' and B/B' pairs is a consequence of conformational equilibrium, whereupon the ethylene bridge attains effective mirror symmetry. The best‐fit vicinal ^3^ *J*‐couplings are 8.8 and 3.9–4.0 Hz in both solvents (Table S2, Figures S18 and S19), indicating similar average conformations of the ethylene bridges. The analysis was extended to compound **3′**, whose spectrum in THF‐*d*_8_ (Figure S20) was well simulated using the best‐fit *J*‐couplings found for **3** in the same solvent (Figure S21). This result strongly suggests that complex **3′** adopts a similar conformation to **3** in THF‐*d*_8_. The same analysis was also performed on the free H_2_bdhb pro‐ligand in both THF‐*d*_8_ and CD_2_Cl_2_ (Table S2, Figures S22 and S23). Here, the two vicinal ^3^ *J*‐couplings (9.5 and 6.0 Hz) are significantly closer to each other, demonstrating that H_2_bdhb has a higher conformational freedom than coordinated bdhb^2−^ in **3** and **3′**.

### 2.4 Conformational Analysis

Nine monomeric species are potentially present in THF solutions of **3**, namely tetracoordinated [Zn(bdhb)], pentacoordinated [Zn(bdhb)(py)] and [Zn(bdhb)(thf)], and hexacoordinated *cis/trans*‐[Zn(bdhb)(py)*_x_* (thf)*_y_*] with *x*+*y* = 2. The low‐lying conformers of these species were calculated using a procedure similar to that described in Ref. [24], relying on an extensive conformational search followed by geometric optimization with the composite DFT approach B97‐3c (see Supporting Information for details). The conformation of the ethylene bridges was invariably found close to *gauche*, with absolute values of the C─CH_2_─CH_2_─C torsion angles spanning a remarkably narrow interval of 61 ± 8° (from 53.3 to 68.3° in the most stable conformer of each species; see Figure S24 and Table S3).

The conformational flexibility of the free bdhb^2−^ ligand was evaluated by DFT on deprotonated 6‐phenylhexane‐2,4‐dione in the gas phase as a model structure. The energy profile for the rotation about the ethylene C─C bond (Figure S25) indicates a free rotation energy barrier of ∼8 kcal mol^−1^ and a rotational barrier to the interconversion of the two *gauche* forms as low as 2–3 kcal mol^−1^.

It is known that the vicinal proton–proton coupling constant (^3^ *J*_HH_) depends on the H─C─C─H dihedral angle, following a Karplus‐type equation such as Equation (4) reported by Altona et al.^[^ ^41^ ^]^ This relationship then allows to predict the ^3^ *J*_HH_ values within the ethylene bridges of bdhb^2‐^ starting from the C─CH_2_─CH_2_─C torsion angle and the empirical group electronegativities (*λ*) of the substituents. The *λ* value for a C(═O)R group was reported as *λ*_1 _= 0.51^[^ ^41^ ^]^ and that of a phenyl ring (*λ*_2 _= 0.5) can be derived from ^3^ *J*_HH_ in isopropylbenzene (mean value 6.9 Hz)^[^ ^42^ ^]^ using Equation (2) reported by Altona et al. and 0.8 as the empirical electronegativity of a methyl group.^[^ ^41^ ^]^ For standard torsion angles of ±60° and 180°, which correspond to the two *gauche* and the *anti* conformers of the ethylene bridge^[^ ^41^ ^]^ (Figure S26), the estimated ^3^ *J*_HH_ values are presented in Table 3. It can be seen that the mean *J* values for an equimolar mixture of the two *gauche* conformers (9.0 and 3.6 Hz, case B) compare well with the experimental values obtained from the analysis of the second‐order multiplets of H*f* and H*e* protons in coordinated bdhb^2−^ (8.8 and 3.9–4.0 Hz). A good accordance between estimated and experimental vicinal coupling constants is found also for the free ligand, considering a molar fraction of the *anti* conformer slightly above 0.5 (5.9 and 9.4 Hz in case C versus experimental values of 6.0 and 9.5 Hz). Note that a prevailing *anti* conformer, as expected for a [Zn_2_(bdhb)_2_(py)_2_] species (Figure 2), would be reflected in a greater difference between the two vicinal coupling constants (^3^ *J*_HH_ around 3.3 and 14.1 Hz).

**Table 3:** Vicinal proton–proton coupling constants (Hz) estimated from Equation (4) in Ref. [41] for the three standard conformers of the ethylene bridge in Figure S26 and for selected mixtures of conformers.

	^3^ *J*_HH_ for Standard Conformers			Mean ^3^ *J*_HH_ for Mixtures of Conformers		
	anti	gauche1	gauche2	case A^[^ ^a^ ^]^	case B^[^ ^b^ ^]^	case C^[^ ^c^ ^]^
H_1_H_4_	3.34	14.08	3.86	7.08	8.97	5.87
H_1_H_3_	14.08	3.86	3.34	7.08	3.60	9.36
H_2_H_3_	3.34	3.86	14.08	7.08	8.97	5.87
H_2_H_4_	14.08	3.34	3.86	7.08	3.60	9.36

^[a]^ Molar fractions: *x*_anti_ = *x*_gauche1_ = *x*_gauche2_ = 0.33;

^[b]^ Molar fractions: *x*_anti_ = 0.0, *x*_gauche1_ = *x*_gauche2_ = 0.5;

^[c]^ Molar fractions: *x*_anti_ = 0.55, *x*_gauche1_ = *x*_gauche2_ = 0.225;

## 3 Conclusion

A diamagnetic analogue of the known paramagnetic complexes [(VO)_2_(bdhb)_2_] (**1**) and [Co_2_(bdhb)_2_(py)_4_] (**2**), namely [Zn_2_(bdhb)_2_(py)_2_] (**3**), was synthesized and comprehensively studied in the crystalline state and in solution. A precursor complex with empirical formula Zn(bdhb)(H_2_O)_2_ (**3′**) was first isolated in solid form and then treated with excess pyridine in THF/*n*‐hexane to give two different crystalline phases belonging to the triclinic (**3*a***) and orthorhombic (**3*o***) crystal systems but sharing very similar molecular structures. Both crystalline phases contain dimeric molecules supported by bridging bdhb^2−^ ligands, as found in **1** and **2**, although the exact conformation of the organic scaffold differs across the series. Having a different number of coordinated pyridine molecules, **3** is also not truly isostructural to the dicobalt(II) complex **2**. Both zinc(II) ions in **3** reside in a square‐pyramidal environment with one axial py ligand, whereas the coordination geometry in **2** is tetragonally elongated octahedral, with two py ligands in the axial positions. Despite these differences, **1**–**3** behave similarly in organic solution and rearrange into quasi‐macrocyclic mononuclear species. Taking advantage of the highly resolved spectra afforded by diamagnetic species, the structural isomerization of **3** in THF‐*d*_8_ and CD_2_Cl_2_ was directly proved by a molecular weight determination using ^1^H DOSY NMR. Conclusions were supported by a *J*‐coupling analysis of the second‐order ^1^H NMR multiplets assigned to the ethylene bridges. The experimental ^3^ *J*_HH_ values compare well with those derived from a Karplus‐type analysis based on the ligand's conformation predicted by DFT calculations. Finally, ESI‐MS spectrometry data provide clear evidence of the presence of both mono‐ and dinuclear species in solution under ESI conditions.

Our results indicate that the adoption of a different structure in the crystalline state and in solution may be a common feature of bdhb^2−^ complexes with divalent transition metal ions. This dual structure is a direct consequence of the utmost conformational flexibility displayed by the acyclic bdhb^2−^ ligand, which can use its two β‐diketonato functions to bind either the same or two different metal ions.

With regards to the design of solid‐state spin qubits, H_2_bdhb and its ring‐closure product 3,5,16,18‐tetraoxo[7.7]metacyclophane^[^ ^21^, ^22^ ^]^ provide opportunities in different directions. The rearrangement of **1** and **3** into monomeric species in solution implies that any attempt to dope crystals of **3** with **1** using co‐crystallization from solution will be affected by metal scrambling. At very low doping levels, the dopants will presumably consist of heterobimetallic (VO^2+^, Zn^2+^) complexes, which hold the potential to be used as individual solid‐state qubits. Increasing doping levels will increase the fraction of divanadyl species, which may serve as pairs of dipolar‐coupled qubits.^[^ ^43^ ^]^

It is anticipated that ring closure of H_2_bdhb will enable greater control over molecular structure, with exclusive formation of monomeric complexes both in solution and in the crystalline state. The preparation of these macrocyclic bis(β‐diketones) is an investigation direction we are currently following to access neutral vanadyl‐based qubits using a single multichelating organic ligand with nuclear spin‐free donor atoms.

## 4 Experimental Section

### Materials and Methods

All chemicals were of reagent grade and used as received unless otherwise noted. MeOH was dried using standard methods^[^ ^44^ ^]^ and then stored over activated 3 Å molecular sieves. THF was pre‐dried over KOH,^[^ ^45^ ^]^ and subsequently distilled from its sodium diphenylketyl solution before use. Dry THF and the deuterated solvents (THF‐*d*_8_, CD_2_Cl_2_, and acetone‐*d*_6_) used in NMR experiments were deoxygenated through three freeze–pump–thaw cycles and (except for acetone‐*d*_6_) stored over activated 4 Å molecular sieves. Pyridine was distilled over KOH (115–116 °C) and stored over KOH pellets prior to use. H_2_bdhb was prepared as described elsewhere.^[^ ^24^ ^]^

Combustion analysis was performed using a ThermoFisher Scientific Flash 2000 analyzer. IR spectra were collected in ATR mode on a JASCO 4700 FT‐IR spectrometer, between 400 and 4000 cm^−1^ and with a resolution of 2 cm^−1^.

The NMR spectra were recorded at 298 K in THF‐*d*_8_, CD_2_Cl_2_, and acetone‐*d*_6_ on an AVANCE400 (400.13 MHz for ^1^H) FT‐NMR spectrometer from Bruker Biospin using 5 mm airtight Young‐valved NMR tubes from Norell to prevent a large entry of water and/or dioxygen over time. Spectra were analyzed and processed using TopSpin (version 4.3.0^[^ ^34^ ^]^). The chemical shifts (*δ*) are expressed downfield versus tetramethylsilane (TMS) as an external standard, setting the residual ^1^H signals of THF‐*d*_8_, CD_2_Cl_2_, and acetone‐*d*_6_ at 1.72 [CH_2_(3,4)], 5.32, and 2.05 ppm, respectively.^[^ ^35^ ^]^ Alternatively, TMS was added as an internal standard. The scalar coupling constants (*J*) are expressed in Hz. ^1^H DOSY NMR spectroscopy measurements on **3** in THF‐*d*_8_ and CD_2_Cl_2_, and on H_2_bdhb in THF‐*d*_8_, were carried out at 400.13 MHz and 298.0 K with a *ledbpgp2s* sequence (Bruker library) using bipolar gradient pulses^[^ ^46^ ^]^ with diffusion time *Δ* = 0.060 s and gradient length *δ* = 1000 µs. The signal decay was fitted with a single exponential function using Bruker Dynamic Center software (version 2.8.3). TMS was added as an internal reference for the normalization of measured diffusion coefficients.^[^ ^39^ ^]^ The absolute errors on estimated *MW*s were calculated as reported by Stalke et al.,^[^ ^47^ ^]^ who showed that these errors are largely determined by the uncertainties in ECC's parameters.

The program DAISY implemented in TopSpin 4.3.0^[^ ^34^ ^]^ was used to fit and simulate the 1D ^1^H NMR spectra (see Supporting Information for details).

ESI‐MS measurements were performed in positive‐ion mode on a 6310A Ion Trap LC‐MS(n) instrument (Agilent Technologies) by direct infusion of freshly prepared THF and CH_2_Cl_2_ solutions. First, spectra were recorded in the pure solvents, then MeCN was added to reach a ∼5:1 v/v THF/MeCN or CH_2_Cl_2_/MeCN ratio and improve the ionization. Additional experiments were carried out in CH_2_Cl_2_/pyridine (∼5:1 v/v).

The powder X‐ray diffractogram was acquired on a Panalytical X'Pert PRO diffractometer (*θ*/*θ* geometry, Cu‐Kα radiation) equipped with an X‐celerator detector. Data collection was performed in the range 2*θ *= 5–50°, in steps of 0.017° at a rate of 200 seconds per step. Powder patterns were simulated with Mercury 2023.1.0^[^ ^48^ ^]^ using a full‐width‐at‐half‐maximum of 0.1° in 2*θ*.

### Synthesis of Zn(bdhb)(H_2_O)_2_ (**3′**)

In a two‐neck round bottom flask (25 mL) equipped with a magnetic stirrer, Zn(OAc)_2_·2H_2_O (820.5 mg, 3.738 mmol) was dissolved in dry MeOH (10 mL) under a dinitrogen atmosphere to yield a colorless solution. In a vial, H_2_bdhb (500.6 mg, 1.656 mmol) was dissolved in dry MeOH (5 mL) under a dinitrogen atmosphere to give an orange solution, which was then added dropwise with a syringe to the solution of the metal salt to yield a yellow solution. After several seconds, a white solid started to precipitate. The suspension was stirred at room temperature under an inert atmosphere for 3 hours. The white precipitate was collected by filtration through a fritted glass funnel (porosity G4), washed with dry MeOH until colorless filtrate, and dried in vacuo, yielding a white powder (568.5 mg, 1.415 mmol, 85.5%).

^1^H NMR (400.13 MHz, THF‐*d*_8_, 298 K): *δ* = 7.12 (s, 1H; H*j*), 7.03 (t, ^3^ *J*(*i*,*h*) = 7.5, 1H; H*i*), 6.86 (d, ^3^ *J*(*h*,*i*) = 7.5, 2H; H*h*), 5.22 (s, 2H; H*c*), 2.78 (m, 4H; H*f*), 2.37 (m, 4H; H*e*), 1.84 ppm (s, 6H; H*a*).

^1^H NMR (400.13 MHz, acetone‐*d*_6_, 298 K): *δ* = 7.12 (t, ^3^ *J*(*i*,*h*) = 7.5, 1H; H*i*), 7.04 (s, 1H; H*j*), 6.97 (d, ^3^ *J*(*h*,*i*) = 7.5, 2H; H*h*), 5.39 (s, 2H; H*c*), 2.81 (m, 4H; H*f*), 2.47 (m, 4H; H*e*), 1.91 ppm (s, 6H; H*a*).

IR (ATR):  $\tilde{\nu }$_max_ = 3364 (m, br), 2958 (w), 2929 (w), 2861 (w), 1585 (s), 1559 (w), 1515 (s), 1489 (m), 1455 (m), 1436 (s), 1404 (s), 1360 (s), 1285 (m), 1263 (m), 1253 (m), 1190 (m), 1170 (w), 1159 (w), 1130 (m), 1086 (w), 1018 (m), 1007 (m), 941 (m), 923 (m), 900 (w), 896 (w), 790 (m), 774 (m), 704 (m), 608 (w), 563 (m), 546 (m), 493 (w), 486 (w), 480 (w), 469 (m), 465 (m), 460 (m), 453 (m), 440 (m), 432 (s), 426 (m), 418 (s), 411 (m), 406 (m) cm^−1^.

ESI‐MS (THF/MeCN ∼5:1 v/v, positive‐ion mode): *m/z* (%): 371.2 (4.4) [Zn(bdhb)Li]^+^, 387.1 (34.1) [Zn(bdhb)Na]^+^, 403.1 (46.7) [Zn(bdhb)K]^+^, 737.2 (6.0) [Zn_2_(bdhb)_2_Li]^+^, 753.1 (100.0) [Zn_2_(bdhb)_2_Na]^+^, 769.1 (77.8) [Zn_2_(bdhb)_2_K]^+^.

Elemental analysis calcd (%) for Zn(bdhb)(H_2_O)_1.8_ (C_18_H_23.6_O_5.8_Zn, 398.17): C 54.30, H 5.97; found: C 54.29, H 5.96.

### Synthesis of [Zn_2_(bdhb)_2_(py)_2_] (**3**)

Zn(bdhb)(H_2_O)_2_ (300.1 mg, 0.7537 mmol) was dissolved in dry THF (28 mL), yielding a yellowish solution, which was stirred for 30 minutes, filtered, and layered with *n*‐hexane (∼45 mL) containing pyridine (366 µL, 4.53 mmol). Small colorless needles formed after approximately one week (235.5 mg, 0.2647 mmol, 70.2%).

^1^H NMR (400.13 MHz, THF‐*d*_8_, 298 K): *δ* = 8.60 (m, 2H; H*o*‐py), 7.73 (m, 1H; H*p*‐py), 7.32 (m, 2H; H*m*‐py), 7.19 (br s, 1H; H*j*), 7.02 (t, ^3^ *J*(*i*,*h*) = 7.6, 1H; H*i*), 6.85 (dd, ^3^ *J*(*h*,*i*) = 7.6, ^4^ *J*(*h*,*j*) = 1.0, 2H; H*h*), 5.20 (s, 2H; H*c*), 2.78 (m, ^3^ *J*(*f*,*e*) = 8.8 and 3.9, 4H; H*f*), 2.38 (m, ^3^ *J*(*e*,*f*) = 8.8 and 3.9, 4H; H*e*), 1.83 ppm (s, 6H; H*a*).

^1^H NMR (400.13 MHz, CD_2_Cl_2_, 298 K): *δ* = 8.65 (m, 2H; H*o*‐py), 7.85 (m, 1H; H*p*‐py), 7.43 (m, 2H; H*m*‐py), 7.16 (br s, 1H; H*j*), 7.15 (t, ^3^ *J*(*i*,*h*) = 7.5, 1H; H*i*), 6.97 (dd, ^3^ *J*(*h*,*i*) = 7.5, ^4^ *J*(*h*,*j*) = 1.7, 2H; H*h*), 5.33 (s, 2H; H*c*), 2.83 (m, ^3^ *J*(*f*,*e*) = 8.8 and 4.0, 4H; H*f*), 2.46 (m, ^3^ *J*(*e*,*f*) = 8.8 and 4.0, 4H; H*e*), 1.95 ppm (s, 6H; H*a*).

^1^H NMR (400.13 MHz, acetone‐*d*_6_, 298 K): *δ* = 8.65 (m, 2H; H*o*‐py), 7.87 (m, 1H; H*p*‐py), 7.45 (m, 2H; H*m*‐py), 7.14 (br s, 1H; H*j*), 7.10 (t, ^3^ *J*(*i*,*h*) = 7.5, 1H; H*i*), 6.95 (dd, ^3^ *J*(*h*,*i*) = 7.5, ^4^ *J*(*h*,*j*) = 1.4, 2H; H*h*), 5.34 (s, 2H; H*c*), 2.84–2.79 (m, 4H; H*f*), 2.47 (m, 4H; H*e*), 1.88 ppm (s, 6H; H*a*).

IR (ATR):  $\tilde{\nu }$_max_ = 3102 (w), 3070 (w), 3052 (w), 3023 (w), 2959 (w), 2923 (w), 2859 (w), 1604 (w), 1582 (s), 1574 (s), 1558 (w), 1515 (s), 1488 (m), 1461 (s), 1443 (s), 1416 (s), 1352 (m), 1285 (m), 1260 (w), 1247 (m), 1219 (m), 1190 (m), 1170 (w), 1156 (w), 1130 (m), 1084 (m), 1068 (m), 1042 (m), 1014 (m), 1009 (m), 941 (m), 918 (m), 887 (w), 787 (m), 771 (m), 759 (m), 701 (s), 667 (m), 650 (m), 638 (m), 614 (w), 604 (m), 572 (w), 559 (m), 517 (m), 490 (w), 478 (w), 471 (w), 457 (m), 453 (m), 445 (m), 435 (m), 430 (m), 419 (s), 413 (m), 406 (m), 401 (m) cm^−1^.

ESI‐MS (THF/MeCN ∼ 5:1 v/v, positive‐ion mode): *m/z* (%): 371.2 (4.6) [Zn(bdhb)Li]^+^, 387.1 (33.6) [Zn(bdhb)Na]^+^, 403.1 (46.7) [Zn(bdhb)K]^+^, 737.2 (5.6) [Zn_2_(bdhb)_2_Li]^+^, 753.1 (100.0) [Zn_2_(bdhb)_2_Na]^+^, 769.1 (78.3) [Zn_2_(bdhb)_2_K]^+^.

Elemental analysis calcd (%) for **3** (C_46_H_50_N_2_O_8_Zn_2_, 889.68): C 62.10, H 5.66, N 3.15; found: C 62.19, H 5.51, N 3.40.

### X‐ray Crystallography

The most suitable crystal of **3*o*** found in the synthetic batch was a colorless rod‐like individual, which turned out to be a two‐domain twin (∼1:1) with almost overlapping reciprocal lattice points. It was soaked in NVH immersion oil (Jena Bioscience), placed on a MiTeGen Microloop (diameter = 150 µm), and mounted on a Bruker‐Nonius X8APEX diffractometer equipped with a Mo‐K*α* generator and an area detector for data collection at room temperature. APEX2 v1.0–22 software^[^ ^49^ ^]^ was used for the acquisition of matrix frames and data collection. Data reduction performed with the SAINT‐Plus v7.06A program^[^ ^49^ ^]^ was followed by multi‐scan absorption correction applied with TWINABS v1.05.^[^ ^49^ ^]^

A crystal of **3*a*** was dipped in NHV immersion oil (Jena Bioscience) and mounted on the goniometer head using a Kapton MiTeGen Microloop. The data collection was performed at the X‐ray diffraction beamline (XRD2) of Elettra Synchrotron (Trieste, Italy)^[^ ^50^ ^]^ using monochromatic wavelength of 0.620 Å and Pilatus 6M hybrid‐pixel area detectors (DECTRIS Ltd., Baden‐Daettwil, Switzerland). The complete dataset was collected at 100 K with a dinitrogen stream supplied by an Oxford Cryostream 700, through the rotating crystal method. The diffraction data were then indexed, integrated, and scaled using XDS.^[^ ^51^ ^]^

The structures were solved using SIR92^[^ ^52^ ^]^ (**3*o***) or by the dual space algorithm implemented in the SHELXT code^[^ ^53^ ^]^ (**3*a***). Full matrix least‐squares refinement on *F*_o_^2^ was based on standard methods using SHELXL‐2018/3,^[^ ^54^ ^]^ and both WINGX v2020.2^[^ ^55^ ^]^ and Olex2^[^ ^56^ ^]^ suites. All nonhydrogen atoms were refined anisotropically, while hydrogen atoms were treated as riding contributors in geometrically idealized positions with isotropic *U* = 1.5*U*_eq_(C) and 1.2*U*_eq_(C) for CH_3_ and other hydrogen atoms, respectively. Due to unresolvable disorder, the displacement ellipsoids of some C and N atoms in both structures were unreasonably elongated. Since rigid‐bond restraints (RIGU) cannot be applied in case of disorder, all C and N atoms were restrained to behave more isotropically using the ISOR card. Small deviations from planarity of one pyridine ring in **3*o*** (N2,C42,C43,C44,C45,C46) were corrected using the FLAT card. The solvent‐accessible voids in **3*o*** (Figure S6) were calculated using PLATON software (version 90622).^[^ ^57^ ^]^ The contribution of disordered solvent in these voids was removed from the data using the command SQUEEZE,^[^ ^58^ ^]^ also implemented in PLATON (version 90622).^[^ ^57^ ^]^ This correction lowered the final *R*1 index for reflections with *I* > 2*σ*(*I*) from 8.77% to 5.45%. More importantly, abnormal distortions of pyridine and phenyl rings disappeared, affording a chemically reasonable molecular structure.

Deposition Numbers 2422285 and 2422286 contain the supplementary crystallographic data for this paper. These data are provided free of charge by the joint Cambridge Crystallographic Data Centre and Fachinformationszentrum Karlsruhe Access Structures service.

## Supporting Information

Crystallographic data, additional figures of the structures, powder diffraction patterns, IR, ^1^H and ^1^H DOSY NMR, and ESI‐MS spectra, analysis of the second‐order NMR multiplets of H*f* and H*e* protons, and details of conformational analysis by ^1^H NMR and DFT. CCDC 2422285 and 2422286. The authors have cited additional references within the Supporting Information.^[^ ^59^, ^60^, ^61^, ^62^, ^63^, ^64^, ^65^, ^66^, ^67^, ^68^, ^69^, ^70^, ^71^ ^]^

## Author Contributions

The manuscript was written with the input of all authors. All authors approved the final version of the manuscript.

## Conflict of Interests

The authors declare no conflict of interest.

## Supporting information



Supporting Information

Supporting Information

## Data Availability

The data supporting this study's findings are available in this article's supplementary material.
