# Supplementary material for: Conclusive Insight into the Coordination Complexes of a Flexible Bis(β‐diketonato) Ligand and Their Phase‐Dependent Structure: A Multi‐Technique Approach
Source: Chemistry. 2025 May 13;31(32):e202500697. doi: 10.1002/chem.202500697 (PMC12144875; doi:10.1002/chem.202500697)
Supplement: Supplementary file 1 — Supporting Information [file CHEM-31-e202500697-s002.docx]

**Conclusive Insight into the Coordination Complexes of a Flexible Bis(β-diketonato) Ligand and their Phase-dependent Structure: a Multi-technique Approach**

Manuel Imperato, Alessio Nicolini, Olga Mironova, Enrico Benassi, Nicola Demitri, Lara Gigli, Adele Mucci, and Andrea Cornia*

**Supporting Information**

Table of contents

[1. X-Ray diffraction S2](#_Toc195468857)

[2. IR and NMR spectroscopy S8](#_Toc195468858)

[3. ESI-MS S14](#_Toc195468859)

[4. Analysis of the second-order NMR multiplets of H*f* and H*e* protons in **3** S15](#_Toc195468860)

[5. Conformational analysis S19](#_Toc195468861)

[6. References S24](#_Toc195468862)

# 1. X-Ray diffraction

**Table S1.** Crystal data and refinement parameters for **3*o*** and **3*a***.

|  | **3*o*** | **3*a*** |
| --- | --- | --- |
| Radiation | Mo-Kα (λ = 0.71073 Å) | Synchrotron (λ = 0.620 Å) |
| Chemical formula | C_46_H_50_N_2_O_8_Zn_2_ | C_46_H_50_N_2_O_8_Zn_2_ |
| Formula weight | 889.62 | 889.62 |
| *T* (K) | 298(2) | 100(2) |
| Crystal size (mm^3^) | 0.288 × 0.050 × 0.041 | 0.10 × 0.05 × 0.02 |
| Crystal system | orthorhombic | Triclinic |
| Space group | *Fddd* | *P*$\bar{1}$ |
| *a* (Å) | 16.3608(13) | 10.472(2) |
| *b* (Å) | 40.701(3) | 13.971(3) |
| *c* (Å) | 53.349(4) | 16.248(3) |
| *α* (deg) | 90 | 79.75(3) |
| *β* (deg) | 90 | 71.76(3) |
| *γ* (deg) | 90 | 68.54(3) |
| *V* (Å^3^) | 35525(5) | 2095.7(9) |
| *Z* | 32 | 2 |
| *ρ*_calcd_ (g cm^−3^) | 1.331 | 1.410 |
| 2*θ*_min_/2*θ*_max_ (deg) | 2.79/50.018 | 2.308/35.386 |
| Reflections collected/independent | 13546/--- | 16987/3976 |
| No. of parameters/restraints | 524/291 | 523/288 |
| *R*1/*wR*2 (all data) | 0.2978/0.1412 | 0.1730/0.2738 |
| *R*1/*wR*2 (*I* ≥ 2*σ*(*I*)) | 0.0545/0.1159 | 0.0880/0.2125 |
| GOF | 0.609 | 0.921 |
| Largest diff. peak/hole (*e*Å^−3^) | 0.47/−0.38 | 0.58/−0.88 |

**Figure S1.** Crystals of **3*a*** obtained by liquid diffusion of *n*-hexane/py (6:1 molar ratio of py to Zn) into a THF solution of **3'**.

**Figure S2.** Crystal of **3*o*** selected for SCXRD.


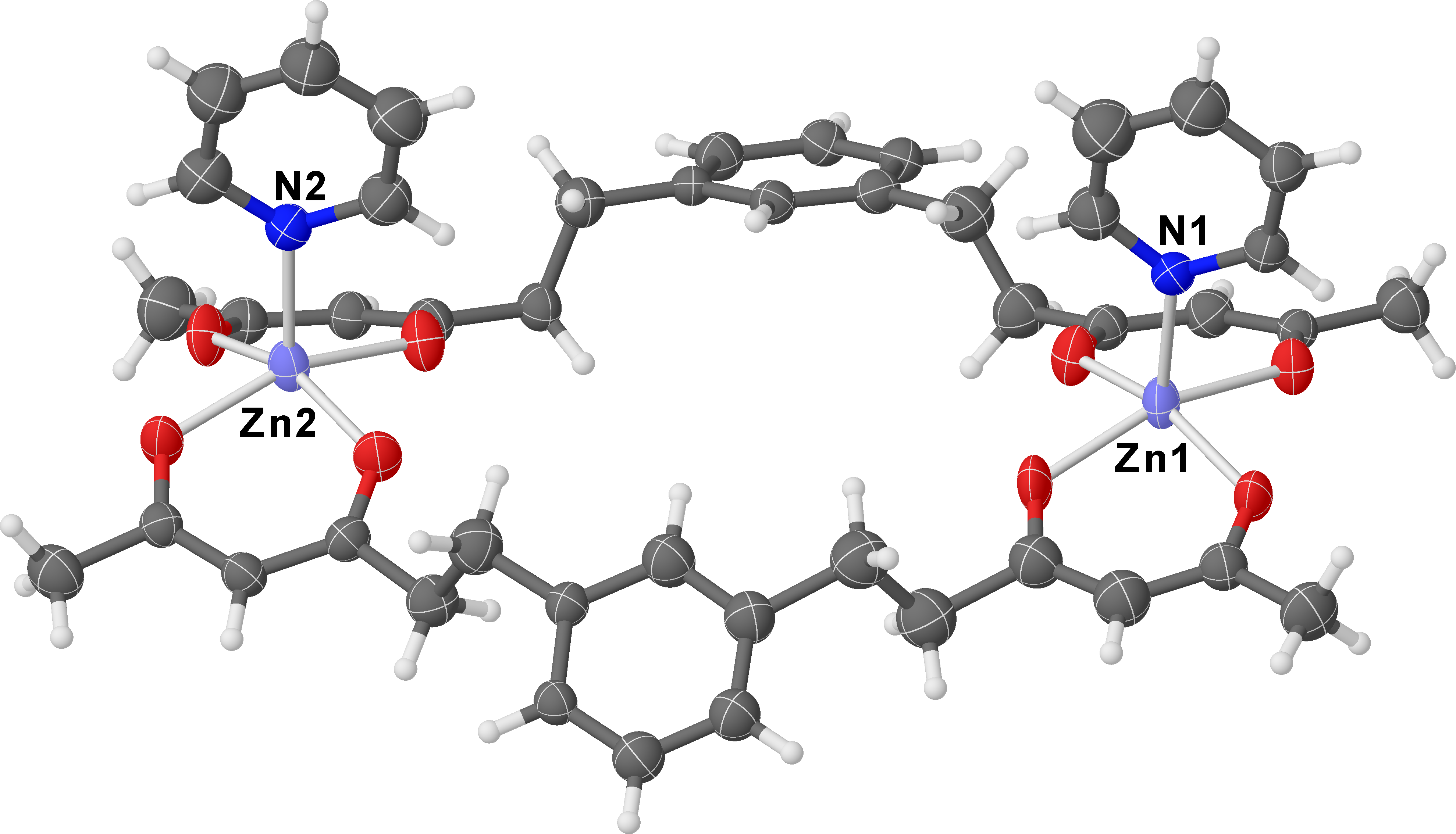


**Figure S3.** Structure of dimeric molecules in **3*o*** with displacement ellipsoids of non-hydrogen atoms drawn at the 40% probability level (Color code: C = dark gray, H = white, O = red, N = blue, Zn = light blue).


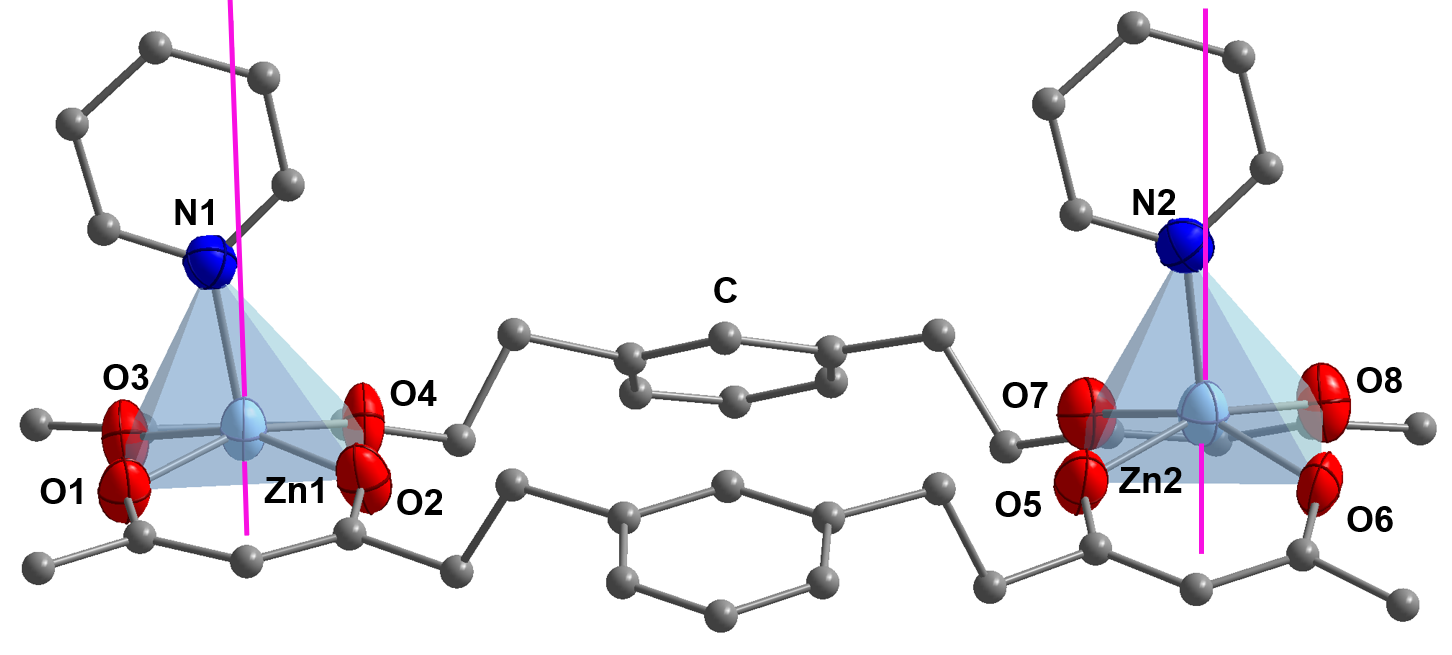


**Figure S4.** Coordination polyhedra of Zn atoms in the structure of **3*o***. The pink lines are the normals to the basal planes of the coordination polyhedra. Zn, O, and N displacement ellipsoids are shown with a 50% probability. Hydrogen atoms are omitted for clarity.


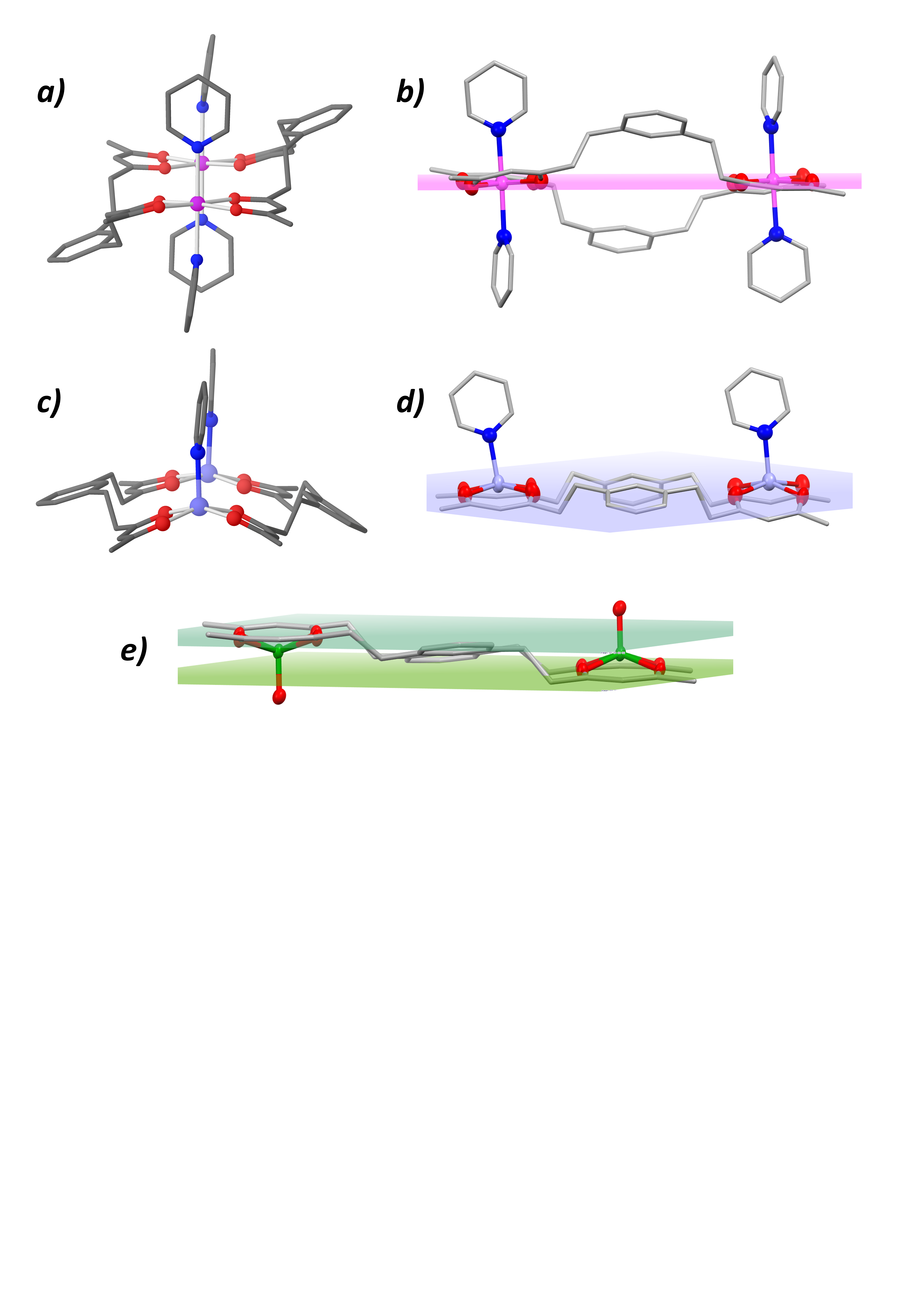


**Figure S5.** Side views of the molecular structures of **2** *(a, b)*, **3*o*** *(c, d)*, and **1** *(e)*. Panels *b)*, *d)*, and *e)* display the mean planes drawn through the β-diketonato O atoms coordinated to the same metal. Hydrogen atoms are omitted for clarity. Color code: C = gray, O = red, N = blue, V = green, Co = pink, Zn = light blue.


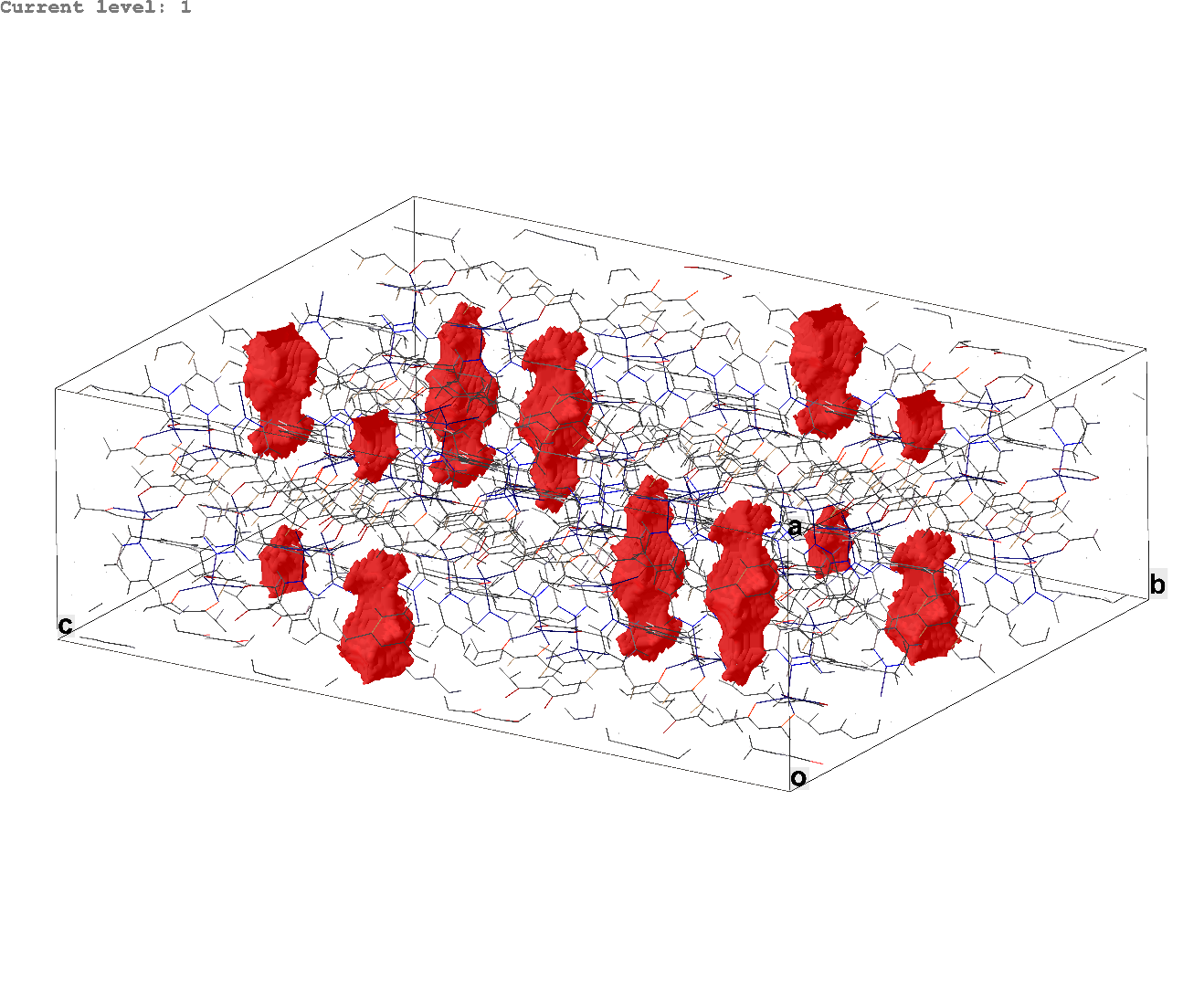


**Figure S6.** Unit cell of **3*o***. The red “blobs” represent the solvent-accessible voids calculated using SQUEEZE.^[1]^

**
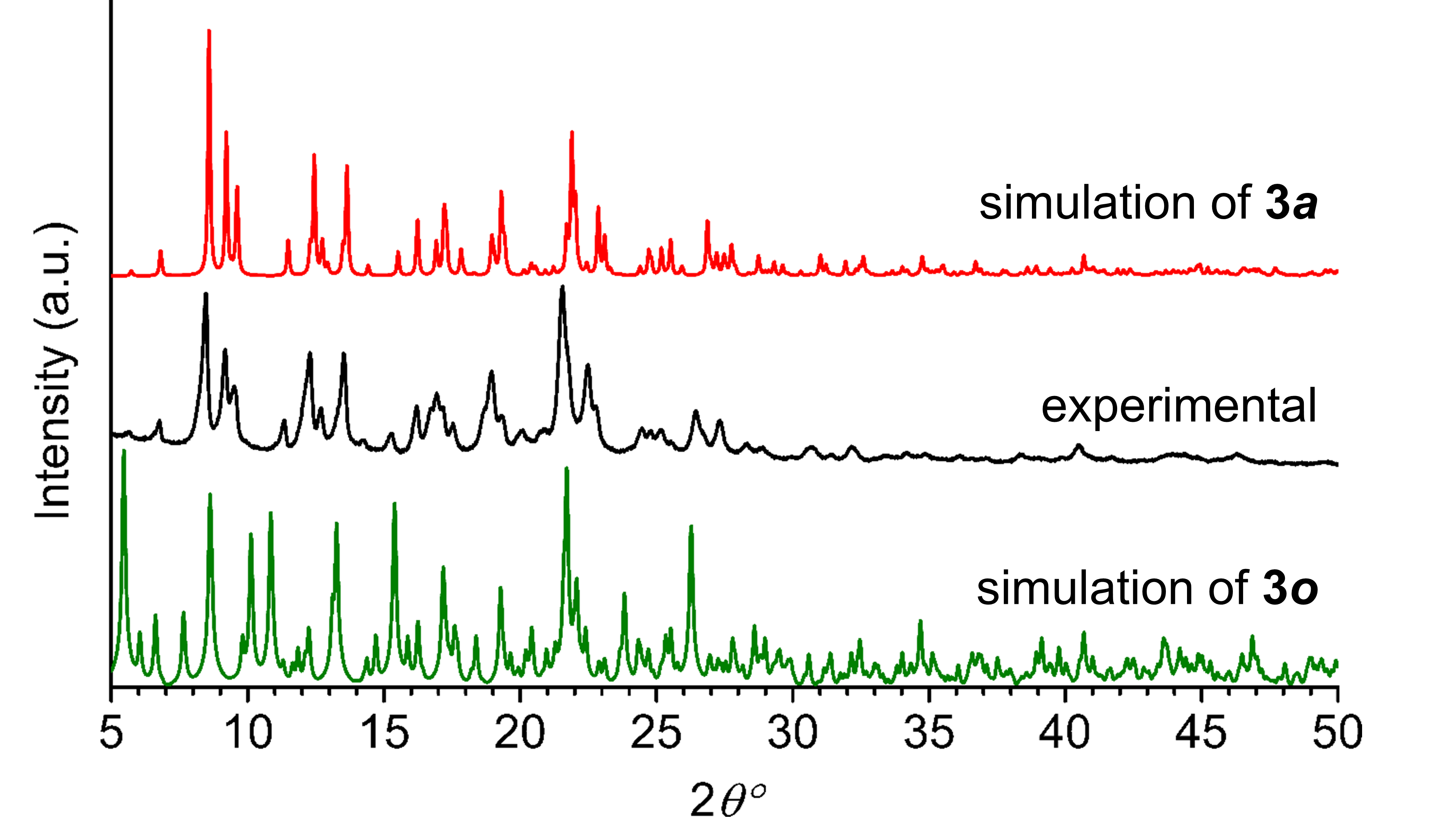
**

**Figure S7.** X-ray powder diffractogram of the bulk microcrystalline product at 298 K (black line). The green and red lines represent the simulated patterns of **3*o*** and **3*a***, respectively. The 2*θ*-shift (+0.13° at 2*θ* = ~8.5°; +0.43° at 2*θ* = ~26.5°) of calculated peak positions for the triclinic phase **3*a*** is due to low-temperature lattice contraction.


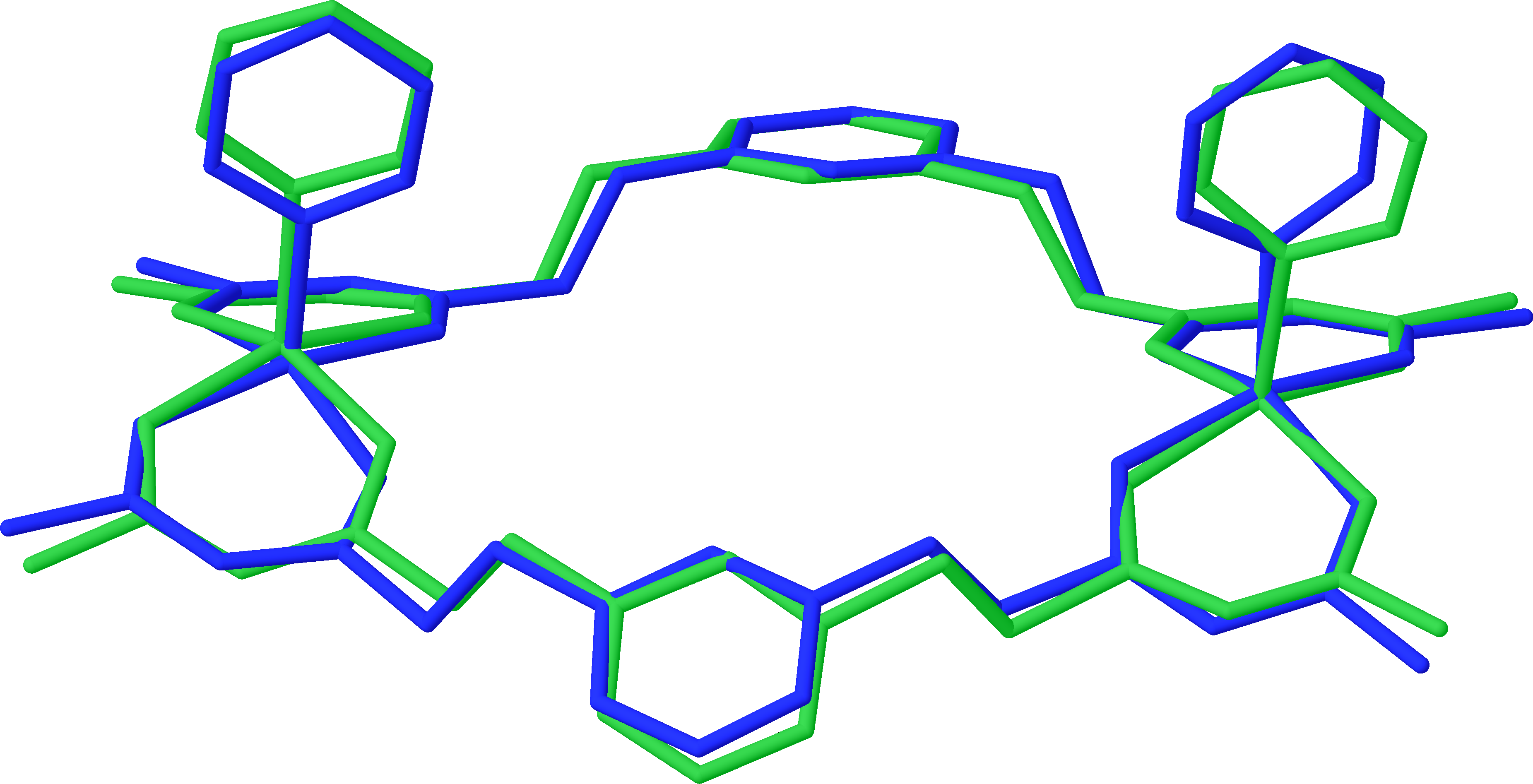


**Figure S8.** Superimposed structures of dimeric complexes in **3*o*** (green) and **3*a*** (blue).

# 2. IR and NMR spectroscopy





**Figure S9.** FT-IR spectra of H_2_bdhb (red), **3'** (blue), and **3*a*** (green).





**Figure S10.** FT-IR spectra of H_2_bdhb (red), **3'** (blue), and **3*a*** (green), zoomed in the region 4000–2000 cm^−1^ (top) and 2000–400 cm^−1^ (bottom).





**Figure S11.** ^1^H NMR spectrum of **3** in CD_2_Cl_2_ (400.13 MHz, 298 K); *δ* = 1.55 (s; H_2_O), 0.00 ppm (s; TMS); processing parameters [TopSpin 4.3.0^[2]^] SI = TD, LB = 0.30 Hz.


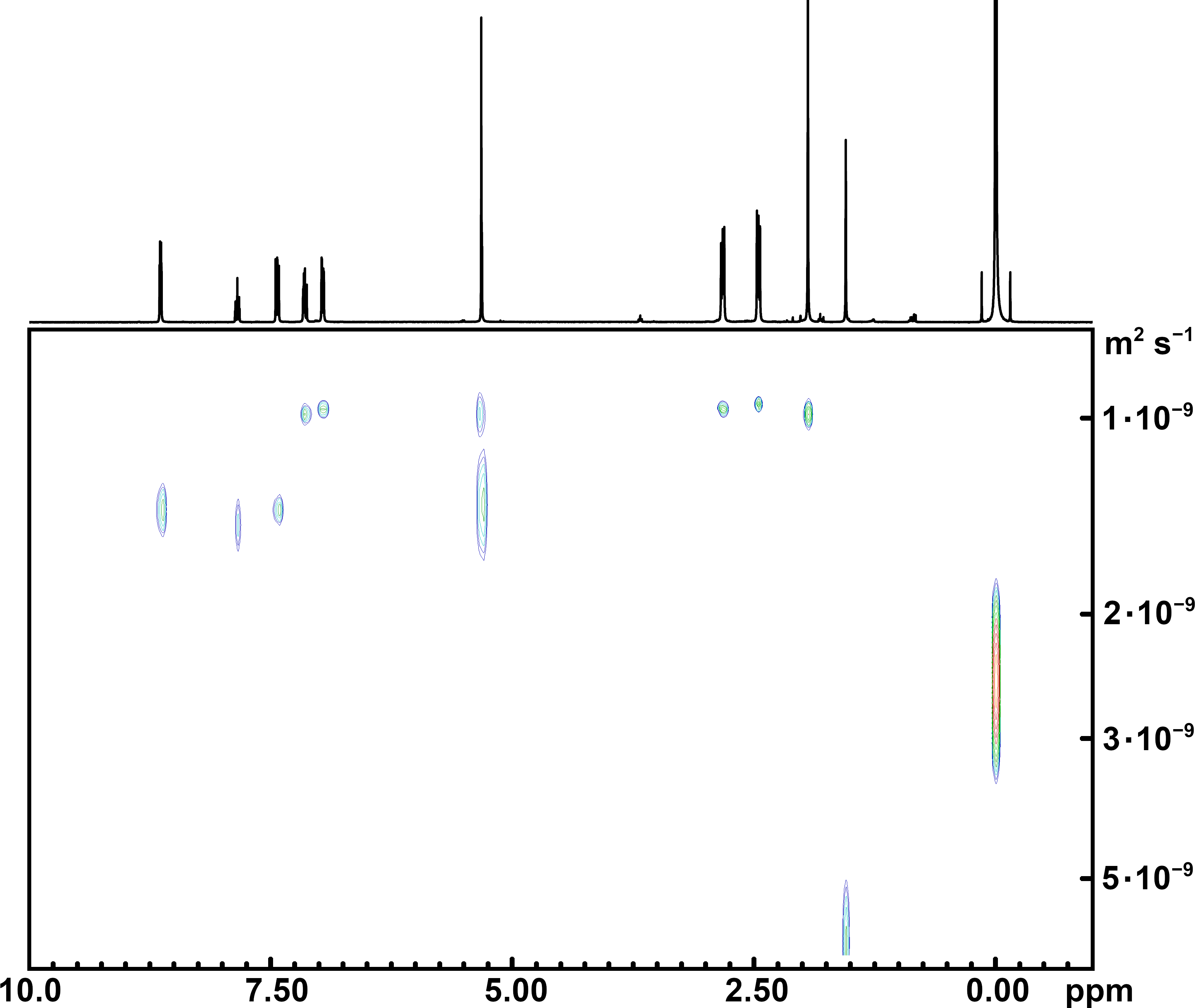


**Figure S12.** ^1^H DOSY NMR spectrum of **3** in CD_2_Cl_2_ (400.13 MHz, 298 K); *δ* = 1.55 (s; H_2_O), 0.00 ppm (s; TMS); processing parameters [TopSpin 4.3.0^[2]^] SI = TD, LB = 0.30 Hz.

**
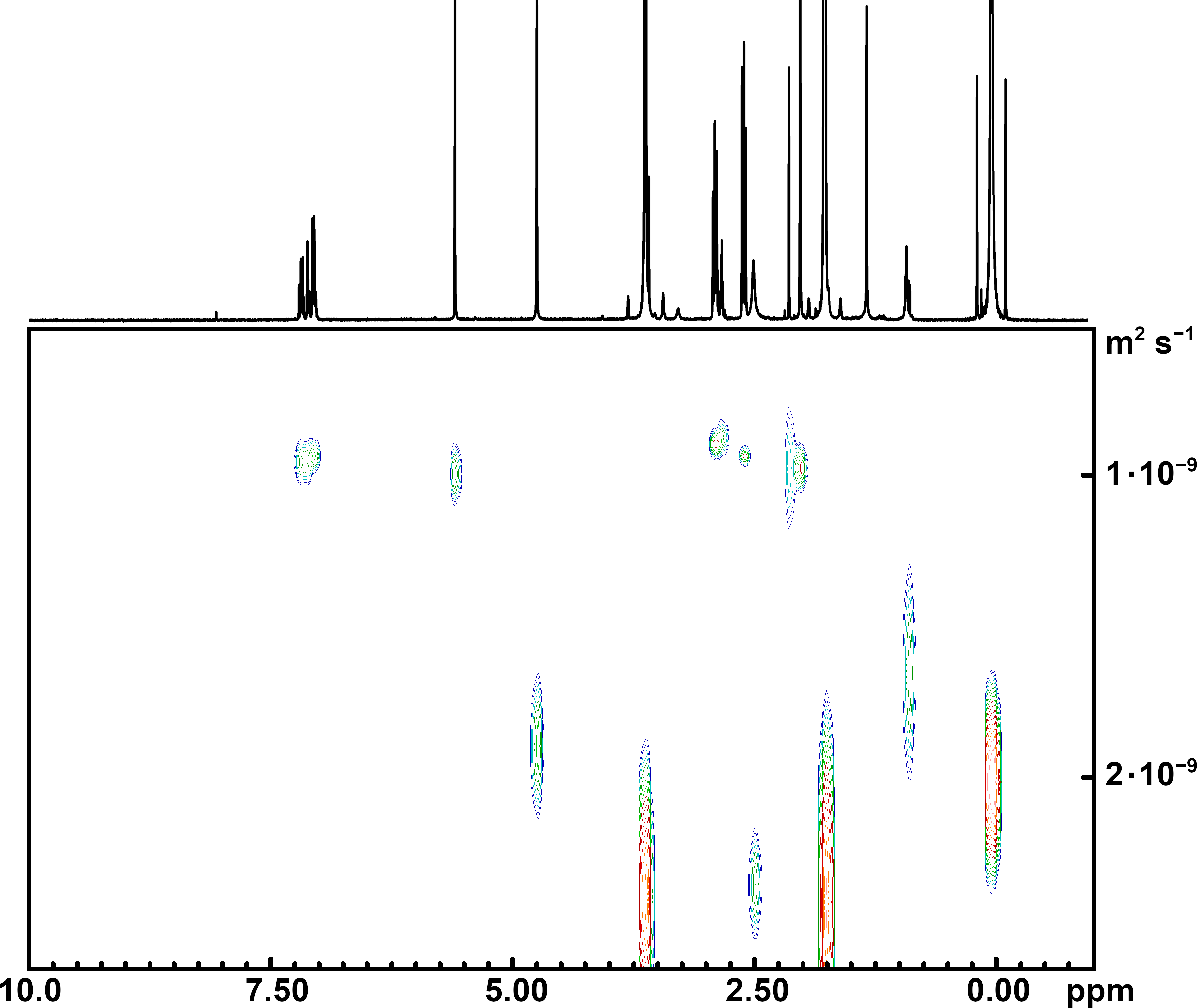
**

**Figure S13.** ^1^H DOSY NMR spectrum of H_2_bdhb in THF-*d*_8_ (400.13 MHz, 298 K); *δ* = 2.46 (s; H_2_O), 1.29 (m; CH_2_, pump oil), 0.88 (m; CH_3_, pump oil), 0.00 ppm (s; TMS); processing parameters (TopSpin 4.3.0^[2]^): SI = TD, LB = 0.30 Hz. The singlet at 4.70 ppm is an unknown impurity in THF-*d*_8_.




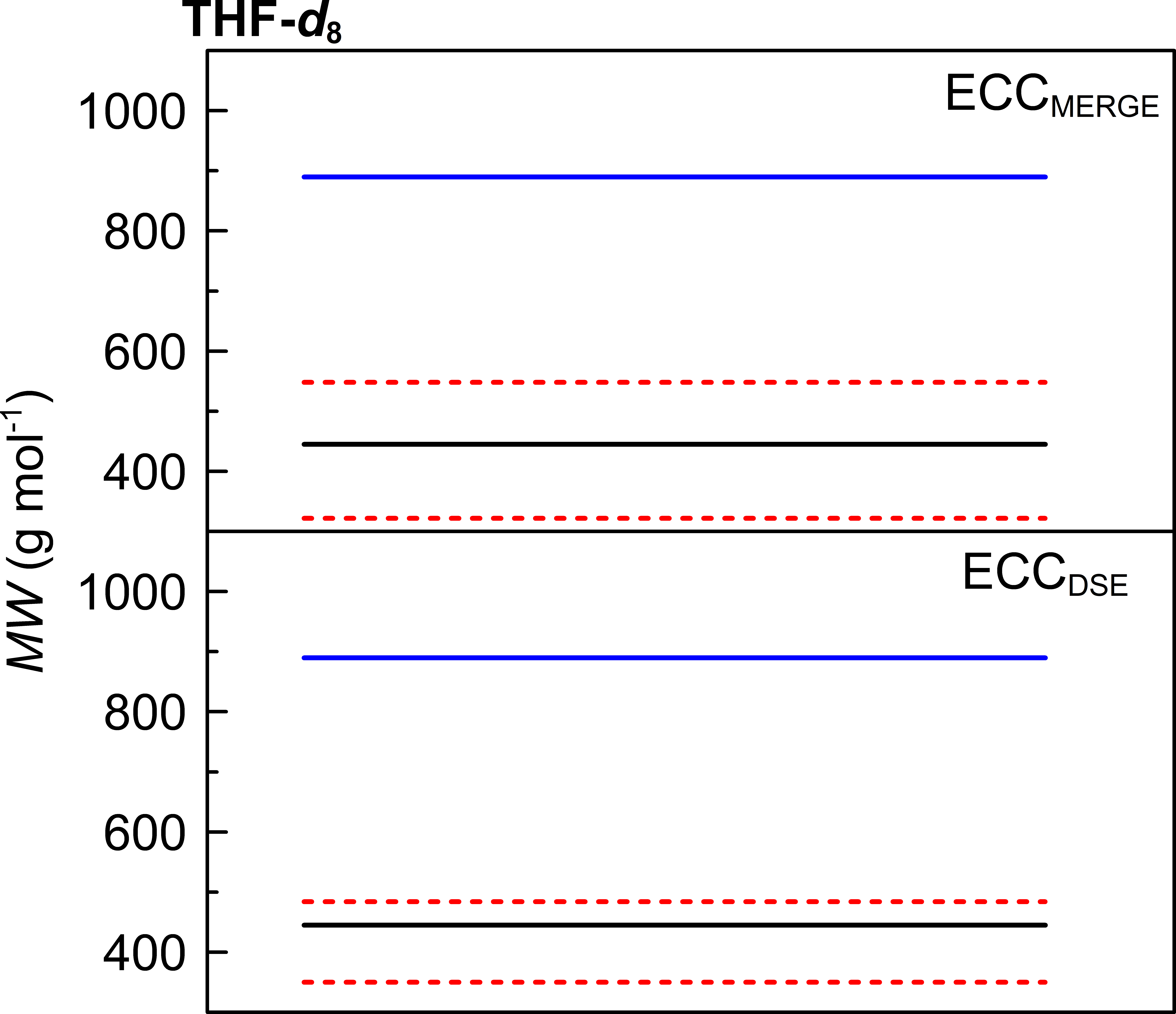


**Figure S14.** Graphical representation of estimated and calculated *MW*s. The dashed red lines represent the upper and lower limits of estimated *MW*s using each ECC (parameters for ECC_DSE_ and ECC_MERGE_ in THF-*d*_8_ and CD_2_Cl_2_ were taken from Ref. ^[3]^ and ^[4]^, respectively. The blue and black lines represent the calculated *MW*s of dimeric **3** and monomeric [Zn(bdhb)(py)], respectively.





**Figure S15.** ^1^H NMR spectra of **1** in CD_2_Cl_2_ (400.13 MHz, 298 K) before (blue line)^[5]^ and after the addition of an approximately equimolar amount of **3** (black lines). The spectrum of the mixture was recorded 5 minutes, 5 days, 16 days, and 7 months after the addition of **3**. The spectrum of **3** in CD_2_Cl_2_ (red line) is also reported for reference. TMS (*δ* = 0.00 ppm, s) was added as internal standard during ^1^H DOSY measurements, which were performed on **1** and **3** before this experiment. Processing parameters [TopSpin 4.3.0^[2]^]: SI = TD, LB = 2.00 Hz.

# 3. ESI-MS


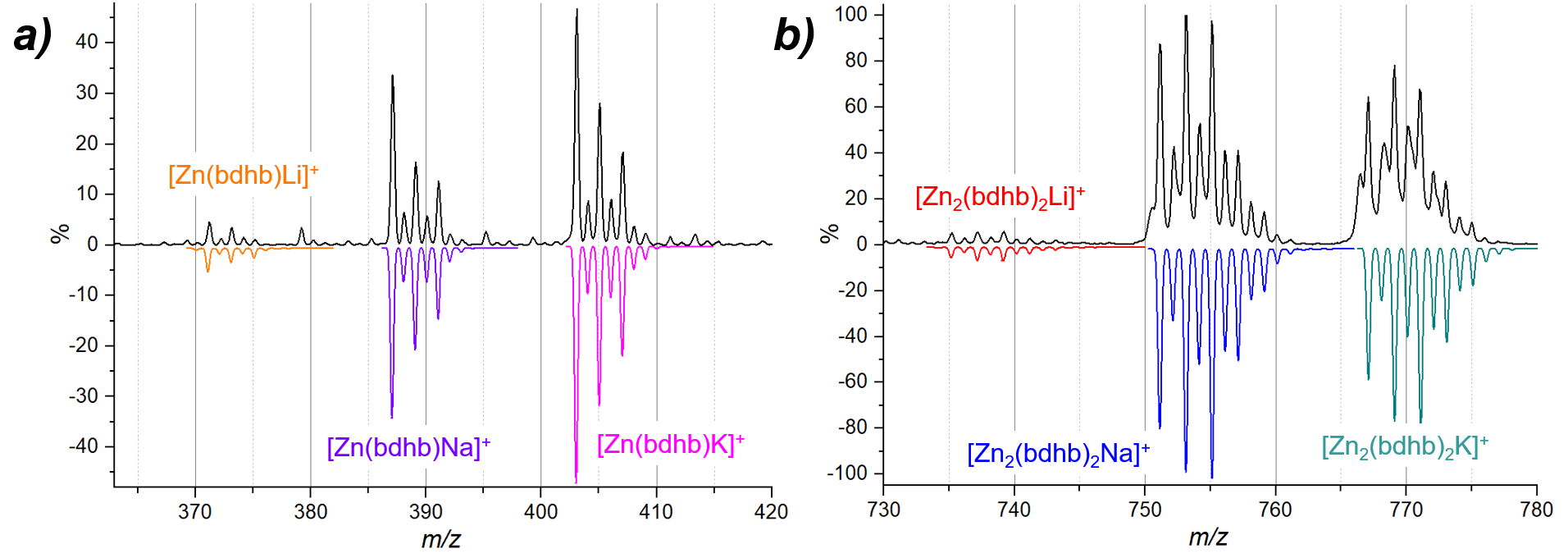


**Figure S16.** Regions of the ESI-MS spectrum of **3** in THF/MeCN (*ca*. 5/1 v/v) showing *a)* monomeric; *b)* dimeric species. The colored lines represent the calculated isotopic patterns.


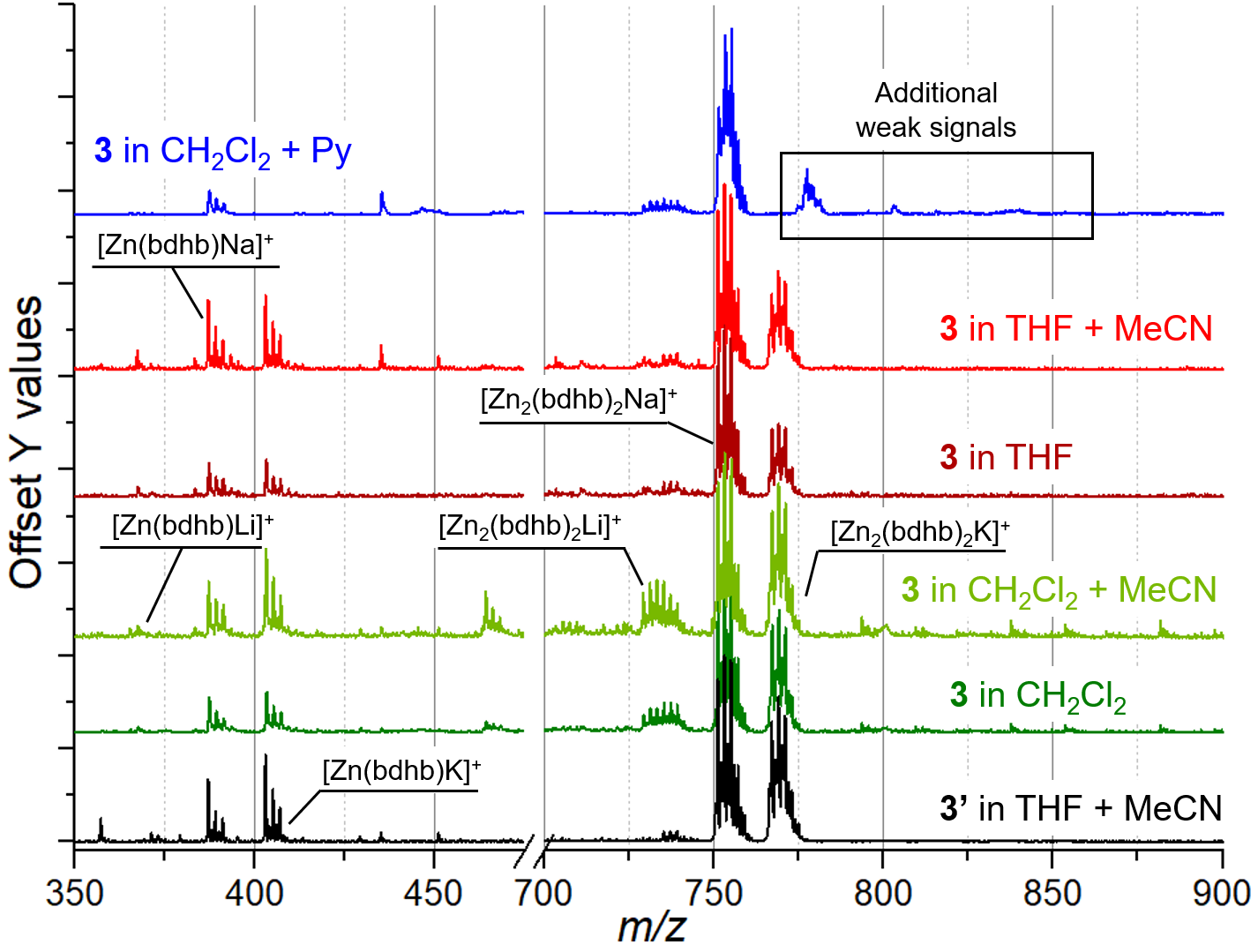


**Figure S17.** Normalized ESI-MS spectra of **3'** in THF:MeCN (~ 5:1 v/v) and of **3** in pure THF, CH_2_Cl_2_, their mixtures with MeCN (~ 5:1 v/v), and a mixture of CH_2_Cl_2_ and py (~ 5:1 v/v), showing the additional weak peaks arising upon addition of excess py.

# 4. Analysis of the second-order NMR multiplets of H*f* and H*e* protons in 3

The −CH_2_−CH_2_− moiety was treated as a simple AA'BB' spin system, where AA' and BB' represent methylene protons H*f* and H*e*, respectively, with ^3^*J*_AB_ = ^3^*J*_A'B'_ and ^3^*J*_AB'_ = ^3^*J*_A'B_. The program DAISY implemented in TopSpin 4.3.0^[2]^ was used to fit and simulate the 1D spectrum. All the resulting parameters are gathered in Table S2.

The high-resolution spectra of **3** in THF-*d*_8_ and in CD_2_Cl_2_ were fitted (Figures S18 and S19) with the Simplex algorithm using all *δ*s, *J*-couplings, and linewidths (LWs) as adjustable parameters. The spectrum of **3'** in THF-*d*_8_ (Figure S20) is less resolved than that of **3** in the same solvent and was simulated using the best-fit *J*-couplings found for **3** in THF-*d*_8_, visually adjusting only *δ*s and LWs (Figure S21). The spectrum of the free pro-ligand (H_2_bdhb) in THF-*d*_8_ was simulated (Figure S22) adjusting all parameters to maximize the visual overlay with the experimental signals assigned to the keto-enolic (KE) branches (this procedure was required since all the algorithms available in DAISY failed to converge to a minimum, due to extensively overlapping signals). The analysis was complicated by the fact that the targeted methylene protons can possess a slightly different *δ* when the neighboring branch is in KE or diketonic (KK) form, as extensively described in Ref.^[6]^ Therefore, two distinct AA'BB' fragments sharing the same *J*s and LWs were considered, with
H*f*_KE-KK_ shifted upfield by 0.006 ppm vs. H*f*_KE-KE_, but the same *δ* for H*e*_KE-KK_ and H*e*_KE-KE_. The relative ratio between the two fragments was fixed to 148:24, in accordance with the occurrence of KE-KE and KE-KK tautomers in 74:24 proportions in THF-*d*_8_.^[6]^ The analysis of the spectrum of H_2_bdhb in CD_2_Cl_2_ was done similarly (Figure S23). Again, two distinct AA'BB' fragments with the same *J*s and LWs were considered, with H*e*_KE-KK_ shifted downfield by 0.003 ppm vs. H*e*_KE-KE_, but the same *δ* for H*f*_KE-KK_ and H*f*_KE-KE_. The relative ratio between the two fragments was fixed to 122:34, since KE-KE and KE-KK tautomers are present in 61:34 proportions in CD_2_Cl_2_.^[6]^

**Table S2.** Chemical shifts (*δ*), *J*-couplings, and linewidths (LWs) resulting from the analysis of H*e* and H*f* NMR signals in **3**, **3'**, and H_2_bdhb (*δ*s are in ppm, while *J*s and LWs are in Hz). AA' and BB' represent H*f* and H*e*, respectively.

| Compound | *δ*_AA'_ | *δ*_BB'_ | ^3^*J*_AB_ = ^3^*J*_A'B'_ | ^3^*J*_AB'_ = ^3^*J*_A'B_ | ^2^*J*_AA'_ | ^2^*J*_BB'_ | LW_AA'_ ^[b]^ | LW_BB'_ ^[b]^ |
| --- | --- | --- | --- | --- | --- | --- | --- | --- |
| **3** in THF-*d*_8_ ^[a]^ | 2.777 | 2.371 | 8.8 | 3.9 | −13.8 | −13.2 | 2.2 | 1.6 |
| **3** in CD_2_Cl_2_ ^[a]^ | 2.827 | 2.458 | 8.8 | 4.0 | −13.9 | −13.9 | 1.9 | 1.5 |
| **3'** in THF-*d*_8_ | 2.781*^c^* | 2.368*^c^* | 8.8 | 3.9 | −13.8 | −13.2 | 3.5*^c^* | 2.9*^c^* |
| H_2_bdhb in THF-*d*_8_ ^[c]^ |  |  |  |  |  |  |  |  |
| *KE-KE fragment* | 2.856 | 2.558 | 9.5 | 6.0 | −13.8 | −12.5 | 1.5 | 1.0 |
| *KE-KK fragment* | 2.850 | 2.558 | 9.5 | 6.0 | −13.8 | −12.5 | 1.5 | 1.0 |
| H_2_bdhb in CD_2_Cl_2_ ^[c]^ |  |  |  |  |  |  |  |  |
| *KE-KE fragment* | 2.883 | 2.575 | 9.5 | 6.0 | −13.8 | −12.5 | 1.8 | 1.0 |
| *KE-KK fragment* | 2.883 | 2.578 | 9.5 | 6.0 | −13.8 | −12.5 | 1.8 | 1.0 |

[a] Best-fit parameters from Simplex algorithm. [b] The systematically higher value of LW_AA'_ compared to LW_BB'_ is related to the long-range *J*-couplings of H*f* with aromatic protons. [c] Parameters providing the best visual agreement with experimental data.





**Figure S18.** Black line: ^1^H NMR spectrum of the methylene protons of **3** in THF-*d*_8_ (400.13 MHz, 298 K); processing parameters [TopSpin 4.3.0^[2]^]: SI = TD, LB = 0.30 Hz. Red line: calculated spectrum using the parameters listed in Table S2.

_

_

**Figure S19.** Black line: ^1^H NMR spectrum of the methylene protons of **3** in CD_2_Cl_2_ (400.13 MHz, 298 K); processing parameters [TopSpin 4.3.0^[2]^]: SI = TD, LB = 0.30 Hz. Red line: calculated spectrum using the parameters listed in Table S2.





**Figure S20.** ^1^H NMR spectrum of **3'** in THF-*d*_8_ (400.13 MHz, 298 K); *δ* = 2.52 (s; H_2_O), 2.48 ppm (s: HOD); processing parameters [TopSpin 4.3.0^[2]^]: SI = TD, LB = 0.30 Hz.

**

**

**Figure S21.** Black line: ^1^H NMR spectrum of the methylene protons of **3'** in THF-*d*_8_ (400.13 MHz, 298 K); processing parameters [TopSpin 4.3.0^[2]^]: SI = TD, LB = 0.30 Hz. Red line: calculated spectrum using the parameters listed in Table S2.





**Figure S22.** Black line: ^1^H NMR spectrum of the methylene protons of H_2_bdhb in THF-*d*_8_ (400.13 MHz, 298 K); processing parameters [TopSpin 4.3.0^[2]^]: SI = TD, LB = 0.30 Hz. Red line: calculated spectrum using the parameters listed in Table S2. It was not possible to analyze the signals of the diketonic (KK) branches due to extensive overlap.

**

**

**Figure S23.** Black line: ^1^H NMR spectrum of the methylene protons of H_2_bdhb in CD_2_Cl_2_ (400.13 MHz, 298 K); processing parameters [TopSpin 4.3.0^[2]^]: SI = TD, LB = 0.30 Hz. Red line: calculated spectrum using the parameters listed in Table S2. It was not possible to analyze the signals of the diketonic (KK) branches due to extensive overlap.

# 5. Conformational analysis

Meta-dynamics conformational searches were based on the semiempirical tight-binding quantum chemistry method GFN2-xTB,^[7]^ as implemented in the conformer-rotamer ensemble sampling program CREST.^[8]^ Structures were assigned a neutral charge and a singlet spin state (*S* = 0), and the generalized Born and solvent accessible surface area model (GBSA) was used to describe implicit solvation by THF. To cover as much conformational space as possible, multiple starting geometries were tested^[9]^ for each of the nine chemical species herein considered, i.e. [Zn(bdhb)], [Zn(bdhb)(py)], [Zn(bdhb)(thf)], *cis/trans*-[Zn(bdhb)(py)_2_], *cis/trans*-[Zn(bdhb)(py)(thf)], and *cis/trans*-[Zn(bdhb)(thf)_2_]. Each starting geometry was pre-optimized by GFN2-xTB with LOOSE convergence criteria before multiple (20) CREST runs were carried out. The ensemble of conformers of each chemical species was sorted to remove duplicates and rotamers based on default parameters (single-point energy window = 6 kcal mol^−1^, RMSD threshold = 0.125 Å, single-point energy threshold between conformer pairs = 0.05 kcal mol^−1^, lower bound for the rotational constant threshold = 0.01). The resulting set of unique conformers was simplified by Principal Component Analysis (PCA) and *k*-means clustering^[10,11]^ to give representative low-energy structures. Each of them was optimized using ORCA 5.0.4^[12–15]^ and the composite DFT approach B97-3c,^[11,16]^ which embeds the atom-pairwise dispersion correction with the Becke-Johnson damping scheme (D3BJ).^[17,18]^ Implicit solvation by THF was described with the Conductor-like Polarizable Continuum Model (CPCM).^[19]^ DEFGRID3, TightSCF, and TightOPT settings were used throughout. After structural optimization, vibrational frequencies were calculated in the harmonic approximation. Structures with negative frequencies, i.e. not corresponding to (local) energy minima, as well as structures displaying an altered connectivity (e.g. ligand dissociation) or a different stereoisomeric form were discarded. Thermochemical data were then computed at 1 atm and 298.15 K, assuming ideal gas behavior. The thermochemical and geometric data for the thermodynamically most stable (i.e. with lowest *G*°) conformers are presented in Table S3.

The energy profile for a full rotation about the ethylene C-C bond in the bhdb^2−^ ligand was evaluated by relaxed surface scans on deprotonated 6-phenylhexane-2,4-dione in the gas phase using ORCA 5.0.4 (same grid and convergence settings as above). Twenty preliminary CREST runs (with default settings) gave an ensemble of 28 unique low-lying conformers, whose energy was re-evaluated by a crude geometry optimization at the BP86/def2-SVP/D3BJ level. The lowest energy conformer was used as the starting point for the subsequent constrained geometry optimization. The torsion angle about the ethylene C-C bond was varied from 70° to 430° (clockwise mode) and from 70° to −290° (counterclockwise mode) in steps of 10° and all the remaining degrees of freedom were relaxed. Figure S25 shows the consistent energy profiles obtained at three different levels of theory.

**Table S3.** Thermochemical and geometric data for the thermodynamically most stable conformers of each chemical species, calculated using the composite DFT approach B97-3c/D3BJ/CPCM(THF).

|  | Final single-point energy (E_h_) | *G*° (E_h_)^[a]^ | C−CH_2_−CH_2_−C  torsion angles (°) | |
| --- | --- | --- | --- | --- |
| [Zn(bdhb)] | -2778.51290957893 | -2778.22192905 | -63.061 | 53.327 |
| [Zn(bdhb)(py)] | -3026.69944025634 | -3026.32627218 | 53.469 | -60.614 |
| [Zn(bdhb)(thf)] | -3010.86934046430 | -3010.47000992 | -54.405 | 59.785 |
| *cis*-[Zn(bdhb)(py)_2_] | -3274.88019382575 | -3274.42487593 | -68.340 | 58.638 |
| *trans*-[Zn(bdhb)(py)_2_] | -3274.87724709448 | -3274.42216406 | -67.259 | 67.292 |
| *cis*-[Zn(bdhb)(py)(thf)] | -3259.05384524292 | -3258.57057178 | -67.312 | 59.160 |
| *trans*-[Zn(bdhb)(py)(thf)] | -3259.05199482235 | -3258.56864612 | -68.076 | 67.805 |
| *cis*-[Zn(bdhb)(thf)_2_] | -3243.22780890164 | -3242.71705689 | -68.123 | 59.681 |
| *trans*-[Zn(bdhb)(thf)_2_] | -3243.22617619885 | -3242.71460053 | -67.502 | 67.283 |

[a] 1 atm and 298.15 K, ideal gas behavior.


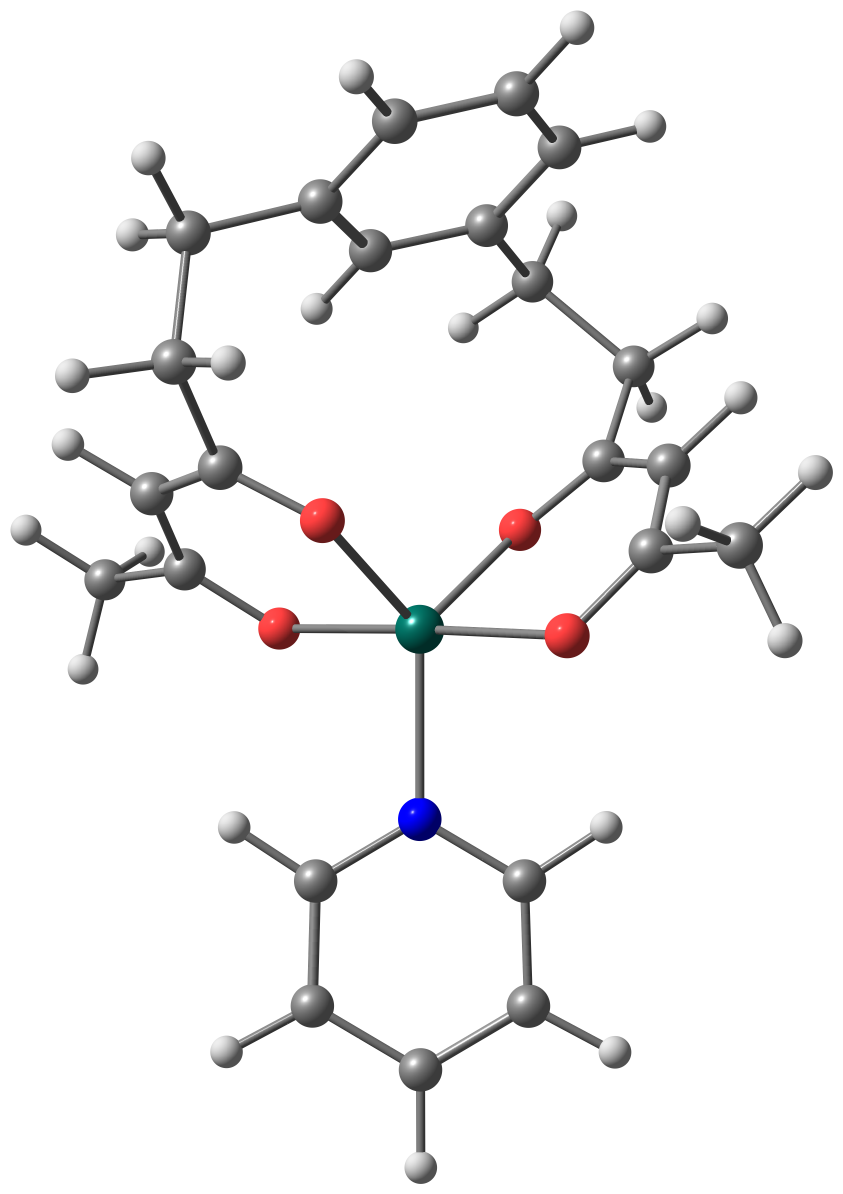


**Figure S24.** Structure of the thermodynamically most stable conformer of [Zn(bdhb)(py)], as resulting from DFT geometry optimization at the B97-3c/D3BJ/CPCM(THF) level. Color code: C = grey, H = light grey, N = blue, O = red, Zn = green.


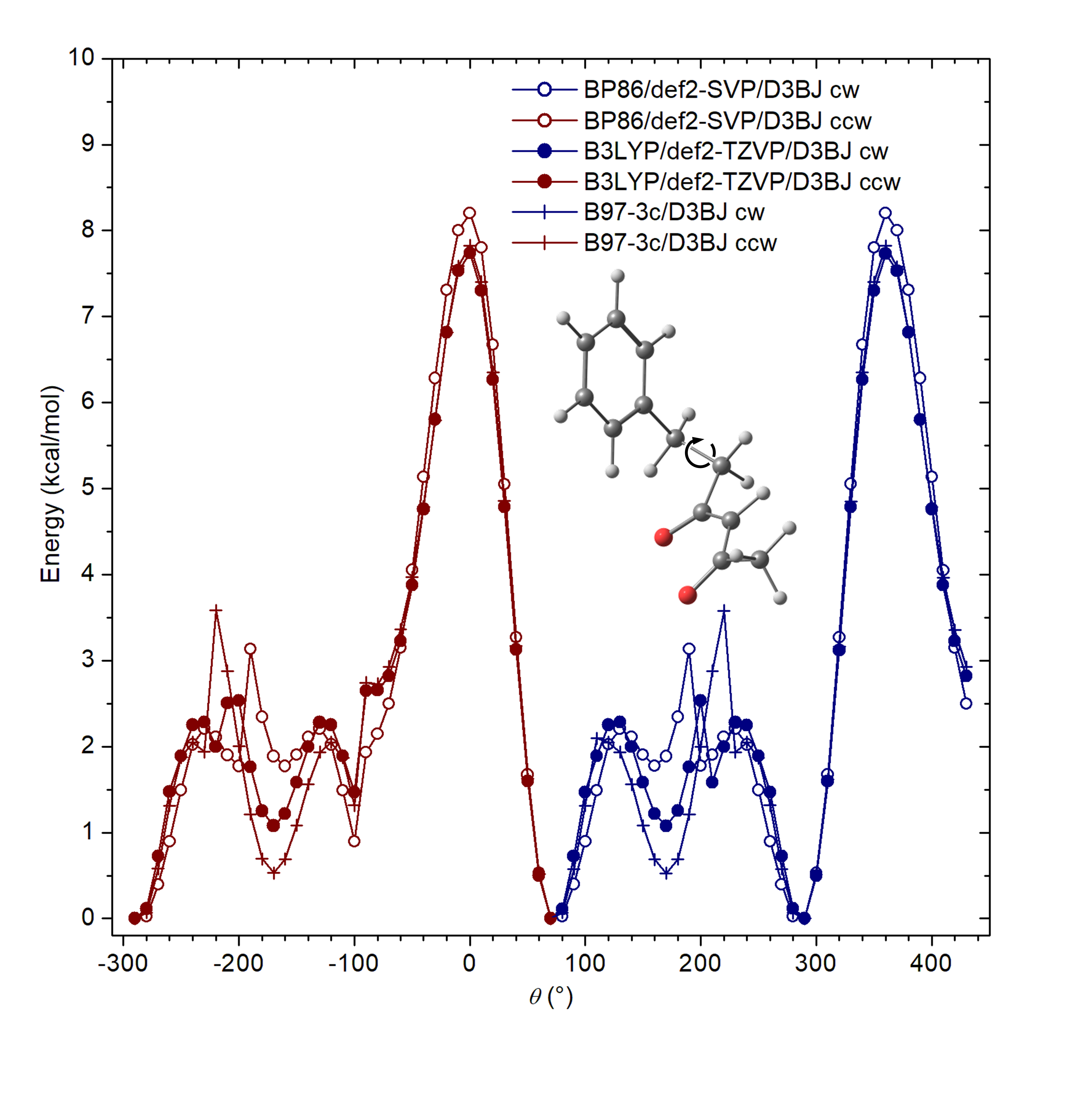


**Figure S25.** Rotational energy profiles resulting from constrained geometry optimization as a function of the torsion angle (*θ*) about the ethylene C−C bond of deprotonated 6-phenylhexane-2,4-dione in the gas phase. The curves were computed at three levels of theory and using both clockwise (cw) and counterclockwise (ccw) rotation starting from *θ* = 70°. Absolute minima correspond to *gauche* conformations of the ethylene bridge.

**Figure S26.** Standard conformers of the ethylene bridge in free H_2_bdhb and in coordinated bdhb^2−^.

# 6. References

[1] A. L. Spek, *Acta Crystallogr. Sect. C Struct. Chem.* **2015**, *71*, 9–18.

[2] *TopSpin 4.3.0*, Bruker AXS Inc., Madison, WI, USA, **2023**.

[3] R. Neufeld, D. Stalke, *Chem. Sci.* **2015**, *6*, 3354–3364.

[4] S. Bachmann, R. Neufeld, M. Dzemski, D. Stalke, *Chem. Eur. J.* **2016**, *22*, 8462–8465.

[5] M. Imperato, A. Nicolini, M. Boniburini, D. Sartini, E. Benassi, M. Chiesa, L. Gigli, Y.-K. Liao, A. Raza, E. Salvadori, L. Sorace, A. Cornia, *Inorg. Chem.* **2024**, *63*, 7912–7925.

[6] M. Imperato, A. Nicolini, M. Boniburini, S. Gómez-Coca, E. Ruiz, F. Santanni, L. Sorace, A. Cornia, *Dalton Trans.* **2024**, *53*, 18762–18781.

[7] S. Grimme, *J. Chem. Theory Comput.* **2019**, *15*, 2847–2862.

[8] P. Pracht, F. Bohle, S. Grimme, *Phys. Chem. Chem. Phys.* **2020**, *22*, 7169–7192.

[9] H. Wang, M. Heger, M. H. Al-Jabiri, Y. Xu, *Molecules* **2022**, *27*, 38.

[10] J. Shao, S. W. Tanner, N. Thompson, T. E. Cheatham, *J. Chem. Theory Comput.* **2007**, *3*, 2312–2334.

[11] M. Bursch, A. Hansen, P. Pracht, J. T. Kohn, S. Grimme, *Phys. Chem. Chem. Phys.* **2021**, *23*, 287–299.

[12] F. Neese, *WIREs Comput. Mol. Sci.* **2012**, *2*, 73–78.

[13] F. Neese, *WIREs Comput. Mol. Sci.* **2022**, *12*, e1606.

[14] F. Neese, *WIREs Comput. Mol. Sci.* **2018**, *8*, e1327.

[15] F. Neese, F. Wennmohs, U. Becker, C. Riplinger, *J. Chem. Phys.* **2020**, *152*, 224108.

[16] J. G. Brandenburg, C. Bannwarth, A. Hansen, S. Grimme, *J. Chem. Phys.* **2018**, *148*, 064104.

[17] S. Grimme, S. Ehrlich, L. Goerigk, *J. Comput. Chem.* **2011**, *32*, 1456–1465.

[18] S. Grimme, J. Antony, S. Ehrlich, H. Krieg, *J. Chem. Phys.* **2010**, *132*, 154104.

[19] V. Barone, M. Cossi, *J. Phys. Chem. A* **1998**, *102*, 1995–2001.
